# Causal Relationship Between Anxiety Disorders and Cancers: A Bidirectional Two‐Sample Mendelian Randomization Study

**DOI:** 10.1002/brb3.71002

**Published:** 2025-10-15

**Authors:** Jingyang Su, Jialin Zhang, Jue Wang

**Affiliations:** ^1^ Department of General Internal Medicine Tongde Hospital Affiliated to Zhejiang Chinese Medical University (Tongde Hospital of Zhejiang Province) Hangzhou China; ^2^ The Second Affiliated Hospital of Zhejiang Chinese Medical University (Xinhua Hospital of Zhejiang Province) Hangzhou China; ^3^ Department of Oncology Hangzhou TCM Hospital of Zhejiang Chinese Medical University (Hangzhou Hospital of Traditional Chinese Medicine) Hangzhou China

**Keywords:** anxiety disorders, cancer, Mendelian randomization study, single‐nucleotide polymorphisms

## Abstract

**Background:**

Anxiety disorders are common psychiatric problems that often accompany cancer. The aim of this study was to investigate the potential causal relationship between anxiety disorders and common cancers.

**Methods:**

We utilized publicly available summary statistics from large‐scale genome‐wide association studies, selecting genetic variant loci associated with anxiety disorders and common cancers as instrumental variables (IVs). These IVs underwent quality control according to three underlying assumptions. The results of the Mendelian randomization (MR) study were analyzed using the inverse variance weighted (IVW) method, along with MR‐Egger regression and weighted median estimation (WME) methods, to evaluate the bidirectional causal relationship between anxiety disorders and common cancers. Additionally, we conducted heterogeneity and multivariate tests to validate the IVW results.

**Results:**

The bidirectional Mendelian randomization study revealed no positive causal relationship between anxiety disorders and common cancers. From a genetic perspective, the IVW analysis demonstrated that anxiety disorders are not associated with an increased risk of developing these common cancers (*p* > 0.05). Reverse MR analysis further revealed no significant causal relationship between common cancers and anxiety disorders (*p* > 0.05).

**Conclusions:**

MR analysis results demonstrated no significant causal relationship between anxiety disorders and common cancers.

## Graphical abstract

A Mendelian randomization study was conducted to analyze the causal relationship between anxiety disorders and common cancers based on its three core assumptions. The results did not find an association between the two.

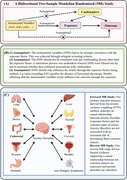

## 1 Introduction

Anxiety is a normal human emotion; however, when it becomes overwhelming and disrupts daily functioning, it warrants clinical attention. Excessive anxiety may result in symptoms such as insomnia, muscle tension, irritability, and gastrointestinal disturbances. Psychiatric rating scales are commonly used to facilitate the diagnosis and assessment of anxiety severity (Locke et al. 2015). Anxiety disorders are among the most prevalent mental health conditions, encompassing generalized anxiety disorder, social anxiety disorder, specific phobias, and so forth. Separation anxiety and selective mutism primarily affect children, while agoraphobia and panic disorder are more common in adulthood (Penninx et al. 2021). Anxiety disorders are not only highly prevalent but also chronic and frequently comorbid with other mental health disorders. Due to their high prevalence, chronic recurrence, and comorbidity with other conditions, the World Health Organization (WHO) ranks anxiety disorders as the ninth leading cause of health‐related disability (Vos et al. 2017). Generalized anxiety disorder has a prevalence of approximately 11.6% in large cohorts of young people (Tiirikainen et al. 2019), while the cumulative prevalence of all anxiety disorders combined ranges from 20% to 30% (Copeland et al. 2014; Ormel et al. 2015), with women being two to three times more likely to be affected than men (Wittchen et al. 2011). Anxiety disorders impose a substantial burden on both individuals and society, accounting for 3.3% of the global disease burden. In Europe alone, the economic cost of anxiety disorders is estimated at 74 billion euros across 30 countries (Gustavsson et al. 2011). This highlights the critical need for effective management of anxiety disorders to enhance individual well‐being and societal health.

Anxiety disorders have a genetic component, with an estimated heritability of 35% in generalized anxiety disorder patients and approximately 50% in social anxiety disorder patients (Meier and Deckert 2019). A meta‐analysis of cohort studies found a significant association between anxiety and cancer incidence and mortality (Wang et al. 2020), and anxiety is frequently comorbid in cancer patients (Kapfhammer 2015). The results of a study based on interviews with oncology patients from different countries found that 10.3% of hematology and oncology patients suffered from anxiety (Mitchell et al. 2011). Another German multicenter cohort study reported a 2.7‐fold increased risk of anxiety in cancer patients compared to the general population (95% CI [2.4–3.1], *p* < 0.001), among cancer types, bladder cancer patients faced the highest risk for elevated anxiety (OR 5.3, 95% CI [3.0–9.4], *p* < 0.001) (Goerling et al. 2023). Meta‐analysis findings further revealed that the prevalence of anxiety in adult cancer patients significantly surpassed that in non‐cancer patients (49.69% vs. 18.37%, OR 6.46, 95% CI [4.36–9.55], *p* = 0.000] (Yang et al. 2013). Cancer‐related symptoms like pain, nausea, and vomiting, influenced by the autonomic nervous system, often exacerbate anxiety and reduce patients' quality of life (Housman et al. 2021). Anxiety may cause behavioral paralysis, hindering patients from using effective coping strategies, impacting medication adherence (Housman et al. 2021), and increasing mortality risk (Linden et al. 2012). Although objective data suggest a causal link between cancer and heightened anxiety risk, the scarcity of high‐quality, low‐bias data presents challenges. Assessing anxiety disorders involves subjective measures, and implementing large randomized controlled clinical studies remains difficult. Therefore, a bidirectional two‐sample Mendelian randomization (MR) study using genome‐wide association study (GWAS) data was performed to investigate potential causal relationships between anxiety disorders and common cancers from a genetic standpoint (Bray et al. 2024). By using genetic variants as instrumental variables, MR studies allow for the examination of causal relationships between exposures and outcomes, improving the reliability of causal inferences (Smith and Ebrahim 2003). Importantly, instrumental variables, such as single‐nucleotide polymorphisms (SNPs), are randomly allocated in MR analysis to minimize confounding and reverse causation.

## 2 Methods

### 2.1 Study Design

In this study, we investigated the potential causal relationship between anxiety disorders and ten common cancers (lung, breast, colorectal, prostate, stomach, liver, thyroid, cervical, bladder, and non‐Hodgkin lymphoma) by examining the pooled dataset from GWAS to identify instrumental variables. We then conducted a bidirectional two‐sample MR study. This involved two stages: a forward MR analysis with anxiety disorders as the exposure factor and the ten common cancers as the outcome, followed by a reverse MR study with the ten common cancers as the exposure factor and anxiety disorders as the outcome.

MR studies rely on three core assumptions:

1. Association assumption: The instrumental variables (SNPs) must exhibit a strong correlation with the exposure factor.
2. Independence assumption: The SNPs should not be correlated with any confounding factors other than the exposure factor.
3. Exclusivity assumption: SNPs should only influence the results through the exposure factors being studied.

### 2.2 Data Sources

Data pertaining to anxiety disorders were sourced from the one Psychiatric Genomics Consortium (PGC) database, comprising 5580 cases and 17,310 controls, all within European populations. Information on ten common cancers was extracted from GWAS summary statistics available in the FinnGen and GWAS databases. Specifically, lung cancer, breast cancer, colorectal cancer, prostate cancer, stomach cancer, thyroid cancer, cervical cancer, bladder cancer, and non‐Hodgkin lymphoma data were obtained from the FinnGen database (https://r12.finngen.fi/). Liver cancer data were acquired from the GWAS database (ID: ieu‐b‐4953), as delineated in Table 1.

**TABLE 1:** The causal effect of anxiety disorders and common cancers.

Exposure	Outcome	IVW			MR‐Egger		Weighted median	
		OR (95% CI)	*p*	FDR (*p*)	OR (95% CI)	*p*	OR (95% CI)	*p*
Anxiety disorders	Lung cancer	1.013 (0.905–1.133)	0.822	0.913	0.874 (0.618–1.236)	0.489	0.874 (0.618–1.236)	0.219
Anxiety disorders	Breast cancer	0.969 (0.913–1.027)	0.287	0.645	0.971 (0.797–1.182)	0.784	0.969 (0.905–1.036)	0.356
Anxiety disorders	Colorectal cancer	0.957 (0.882–1.037)	0.284	0.645	0.926 (0.707–1.213)	0.608	0.970 (0.889–1.058)	0.491
Anxiety disorders	Prostate cancer	1.016 (0.932–1.108)	0.721	0.901	0.844 (0.677–1.052)	0.206	0.964 (0.891–1.043)	0.362
Anxiety disorders	Stomach cancer	1.092(0.936–1.273)	0.262	0.645	1.010 (0.609–1.677)	0.970	1.074 (0.881–1.310)	0.482
Anxiety disorders	Liver cancer	0.917 (0.729–1.153)	0.458	0.654	1.110 (0.560–2.200)	0.780	0.962 (0.723–1.280)	0.788
Anxiety disorders	Thyroid cancer	1.007 (0.883–1.149)	0.915	0.915	0.968 (0.639–1.468)	0.887	0.990 (0.833–1.176)	0.907
Anxiety disorders	Cervical cancer	1.067 (0.928–1.228)	0.361	0.645	0.883 (0.578–1.348)	0.595	0.971 (0.825–1.143)	0.724
Anxiety disorders	Bladder cancer	1.076 (0.969–1.194)	0.170	0.645	0.996 (0.727–1.365)	0.982	1.042 (0.908–1.196)	0.555
Anxiety disorders	Non‐Hodgkin lymphoma	1.051 (0.939–1.177)	0.387	0.645	1.046 (0.717–1.526)	0.828	1.052 (0.922–1.200)	0.454
Lung cancer	Anxiety disorders	0.989 (0.726–1.347)	0.942	0.966	0.993 (0.386–2.554)	0.989	0.951 (0.761–1.189)	0.660
Breast cancer	Anxiety disorders	0.936 (0.856–1.023)	0.145	0.966	0.918 (0.730–1.154)	0.468	0.893 (0.779–1.024)	0.104
Colorectal cancer	Anxiety disorders	1.019 (0.893–1.163)	0.775	0.966	1.069 (0.661–1.729)	0.790	0.991(0.823–1.192)	0.921
Prostate cancer	Anxiety disorders	1.006 (0.937–1.079)	0.876	0.966	0.918 (0.775–1.086)	0.322	0.958 (0.856–1.071)	0.450
Thyroid cancer	Anxiety disorders	0.958 (0.876–1.047)	0.345	0.966	1.021 (0.785–1.328)	0.879	0.969 (0.868–1.082)	0.576
Cervical cancer	Anxiety disorders	0.918 (0.721–1.169)	0.49	0.966	0.970 (0.305–3.087)	0.962	0.943 (0.707–1.258)	0.689
Bladder cancer	Anxiety disorders	0.995 (0.798–1.242)	0.966	0.966	1.360 (0.195–9.495)	0.772	1.014 (0.777–1.324)	0.918
Non‐Hodgkin lymphoma	Anxiety disorders	1.052 (0.882–1.255)	0.573	0.966	1.130 (0.388–3.295)	0.829	1.039 (0.824–1.310)	0.747

Abbreviations: FDR, false discovery rate; *p*, p value.

### 2.3 Extraction of Instrumental Variables

Forward MR analysis was conducted with anxiety disorders as the exposure and ten common cancers (lung, breast, colorectal, prostate, stomach, liver, thyroid, cervical, bladder, and non‐Hodgkin lymphoma) as outcomes. For reverse MR analysis, the exposure–outcome roles were inverted: common cancers served as exposures, and anxiety disorders were the outcome.

SNPs were selected using rigorous criteria to ensure independence and validity: (1) Genome‐wide significance for association with the exposure (*p* < 5 × 10^−8^); however, due to the limited sample size of the anxiety disorders cohort, the threshold was relaxed to *p* < 5 × 10^−6^ to retain sufficient valid SNPs. (2) Linkage disequilibrium (LD) analysis was performed using a 10 Mb genetic distance, and SNPs with high correlation (*r* ^2^ > 0.001) were excluded to minimize LD‐induced bias. (3) SNPs located in the major histocompatibility complex region were omitted to avoid confounding by the complex genetic architecture of this region.

**FIGURE 1:**
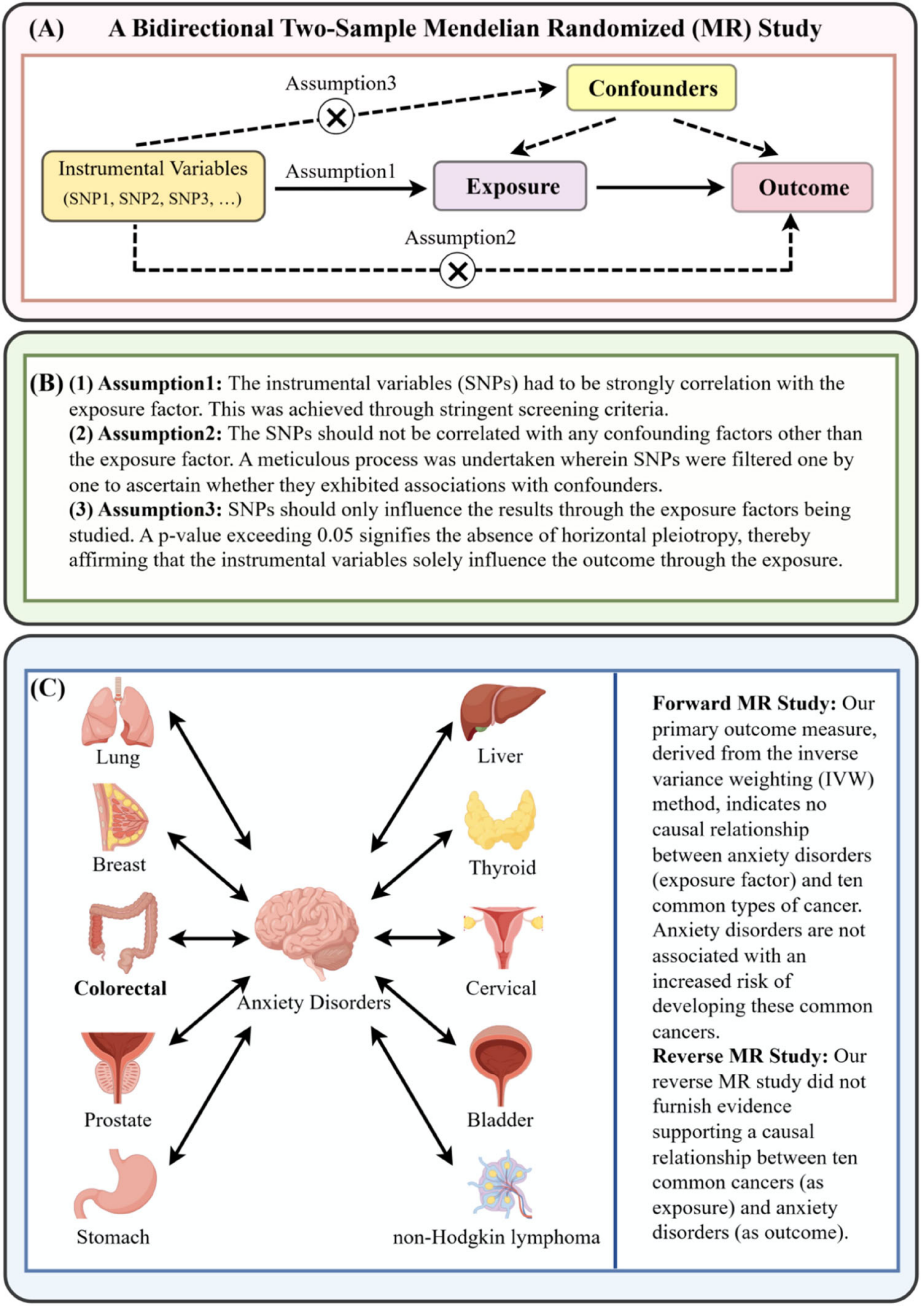
(A) Mendelian randomization principle. (B) Three basic assumptions of Mendelian randomization. (C) Schematic diagram of bidirectional Mendelian randomization of anxiety disorders and common cancers. IVW, inverse variance weighted, MR, Mendelian randomization, SNPs, single‐nucleotide polymorphisms.

Three core assumptions underpin the validity of the MR design, all of which were validated in this study: (1) Strong instrument (Assumption 1): The *F*‐statistic (*F* = beta^2^/SE^2^) for each SNP's association with the exposure was computed, and only SNPs with *F* > 10 were retained (S. Burgess and Thompson 2011; Pierce et al. 2011). (2) No confounding (Assumption 2): Selected SNPs were cross‐checked against published literature, GWAS summary statistics, and the PhenoScanner database to confirm no significant association with potential confounders (e.g., gender, smoking status, body mass index [BMI], education level, depression/neuroticism) (Bowden and Holmes 2019) (3) No horizontal pleiotropy (Assumption 3): The MR‐Egger regression intercept test was used to assess horizontal pleiotropy. A *p* > 0.05 for the intercept indicated no significant pleiotropy, as summarized in Table 2 (S. Burgess and Thompson 2017). Both forward MR studies and reverse MR studies are subject to the above requirements, as shown in Figure 1A,B.

**TABLE 2:** Horizontal pleiotropy in anxiety disorders and common cancers.

Exposure	Outcome	Heterogeneity test				Pleiotropy test		
		IVW		MR‐Egger		MR‐Egger		
		*Q*	*Q*_pval	*Q*	*Q*_pval	Intercept	SE	*p*
Anxiety disorders	Lung cancer	11.387	0.044	9.524	0.049	0.028	0.032	0.426
Anxiety disorders	Breast cancer	7.055	0.217	7.054	0.133	0.000	0.018	0.980
Anxiety disorders	Colorectal cancer	7.271	0.201	7.161	0.128	0.006	0.025	0.816
Anxiety disorders	Prostate cancer	11.208	0.047	6.364	0.174	0.036	0.020	0.156
Anxiety disorders	Stomach cancer	5.425	0.366	5.291	0.259	0.015	0.047	0.766
Anxiety disorders	Liver cancer	1.139	0.951	0.803	0.938	−0.037	0.064	0.593
Anxiety disorders	Thyroid cancer	4.525	0.477	4.482	0.345	0.008	0.039	0.854
Anxiety disorders	Cervical cancer	7.252	0.203	5.956	0.202	0.037	0.039	0.404
Anxiety disorders	Bladder cancer	2.413	0.790	2.155	0.707	0.015	0.029	0.638
Anxiety disorders	Non‐Hodgkin lymphoma	6.582	0.254	6.581	0.160	0.001	0.035	0.979
Lung cancer	Anxiety disorders	9.548	0.049	9.548	0.023	−0.001	0.075	0.993
Breast cancer	Anxiety disorders	39.545	0.930	39.513	0.915	0.002	0.012	0.858
Colorectal cancer	Anxiety disorders	12.072	0.883	12.032	0.846	−0.006	0.028	0.844
Prostate cancer	Anxiety disorders	55.905	0.554	54.534	0.568	0.012	0.011	0.247
Thyroid cancer	Anxiety disorders	14.692	0.197	14.322	0.159	−0.016	0.031	0.622
Cervical cancer	Anxiety disorders	3.238	0.519	3.229	0.358	−0.010	0.107	0.930
Bladder cancer	Anxiety disorders	1.731	0.885	1.630	0.803	−0.041	0.130	0.767
Non‐Hodgkin lymphoma	Anxiety disorders	8.970	0.345	8.948	0.256	−0.010	0.072	0.897

Abbreviation: *Q*, Cochran's *Q*.

### 2.4 Statistical Analysis

After harmonizing the effect alleles of the exposure factors with the outcomes, two‐way MR analysis was conducted employing the inverse variance weighted (IVW) test, MR‐Egger regression, and weighted median estimation (WME). The IVW results served as the primary findings, while MR‐Egger and WMD served as supplementary outcomes, with significance set at *p* < 0.05 (Bowden et al. 2016). In interpreting the potential causal relationship between exposure factors and outcome indicators, odds ratios (OR) and corresponding 95% confidence intervals (CI) were utilized. An OR value > 1 signified that exposure factors might elevate the risk of outcome indicators, with a higher value indicating a relatively higher risk coefficient. To mitigate bias stemming from horizontal pleiotropy, we employed the “mr_pleiotropy_test,” where only a *p* > 0.05 indicated the absence of horizontal pleiotropy, rendering the results more reliable. Similarly, the stability and reliability of results were assessed by examining the MR‐Egger intercept, with results closer to 0 deemed more stable. Furthermore, to gauge the robustness of findings, the Cochran's *Q* statistic was utilized to evaluate the heterogeneity of IVW. A *p* > 0.05 indicated homogeneity, ensuring the reliability of the results (S. Burgess et al. 2013; Bowden et al. 2019). For IVW analyses exhibiting heterogeneity, we applied a random‐effects model to adjust study weights, minimizing the confounding effect of heterogeneity on pooled estimates. Forest plots, scatter plots, leave‐one‐out plots, and funnel plots were also generated to reinforce the data and identify outliers that could potentially influence the MR analysis.

The MR analysis was conducted using the R package “TwoSampleMR” (version 0.6.18) within the R software environment (version 4.4.0).

## 3 Results

### 3.1 MR Analysis

After a series of comprehensive quality controls, we obtained genetic data for anxiety disorders and common cancers for MR analysis, as shown in Figure 1). First, we integrated the anxiety‐related dataset with information on ten common cancers to retain matching SNPs for analysis. In the merged dataset, each dataset contributed six SNPs. Notably, all SNPs exhibited *F*‐statistics > 10, indicating robust instrument strength and enhancing the reliability of our findings, as delineated in Table S2.

Conversely, in reverse MR analysis (where common cancers served as exposures and anxiety disorders as the outcome), we generated eight integrated datasets. However, data analysis for gastric and liver cancers was not feasible due to insufficient eligible SNPs. After aligning datasets for lung cancer, cervical cancer, and anxiety disorder and removing duplicate SNPs, five SNPs were retained per dataset. Breast cancer yielded 55 SNPs, colorectal cancer 20 SNPs, prostate cancer 59 SNPs, thyroid cancer 12 SNPs, bladder cancer 6 SNPs, and non‐Hodgkin lymphoma 9 SNPs. All SNPs exhibited *F*‐values > 10, further validating the robustness of our results, as detailed in (Tables S3–S10).

**FIGURE 2:**
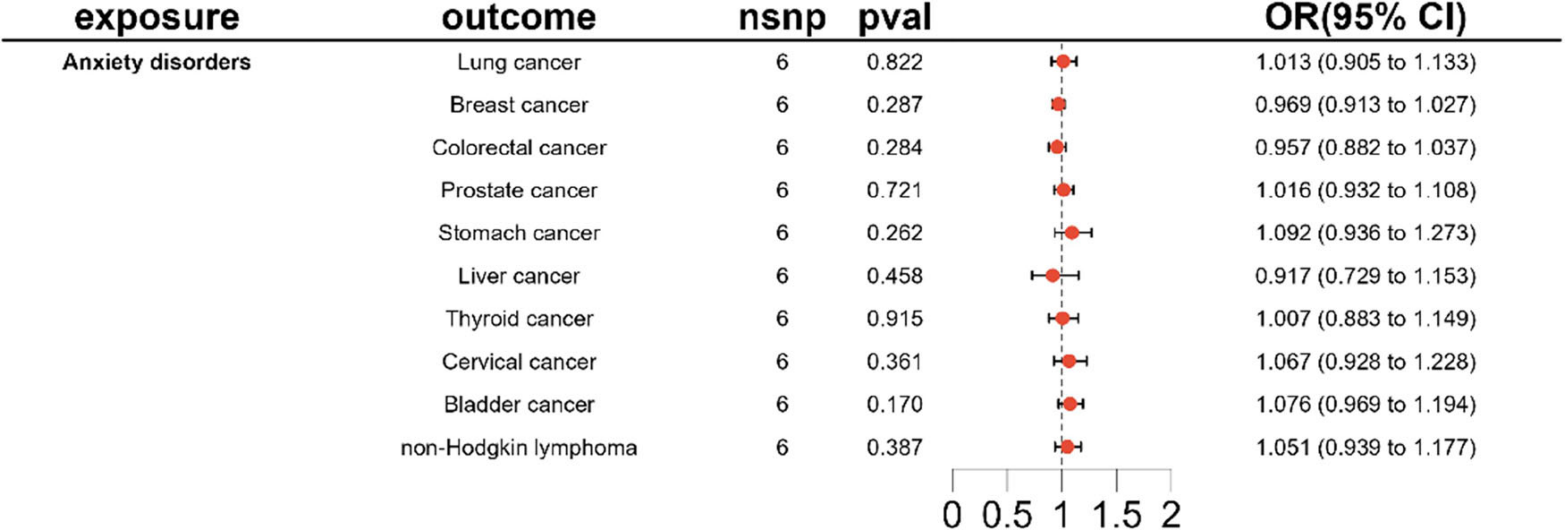
IVW results from a forward Mendelian randomization study with anxiety disorder as an exposure factor common cancer as an outcome.

**FIGURE 3:**
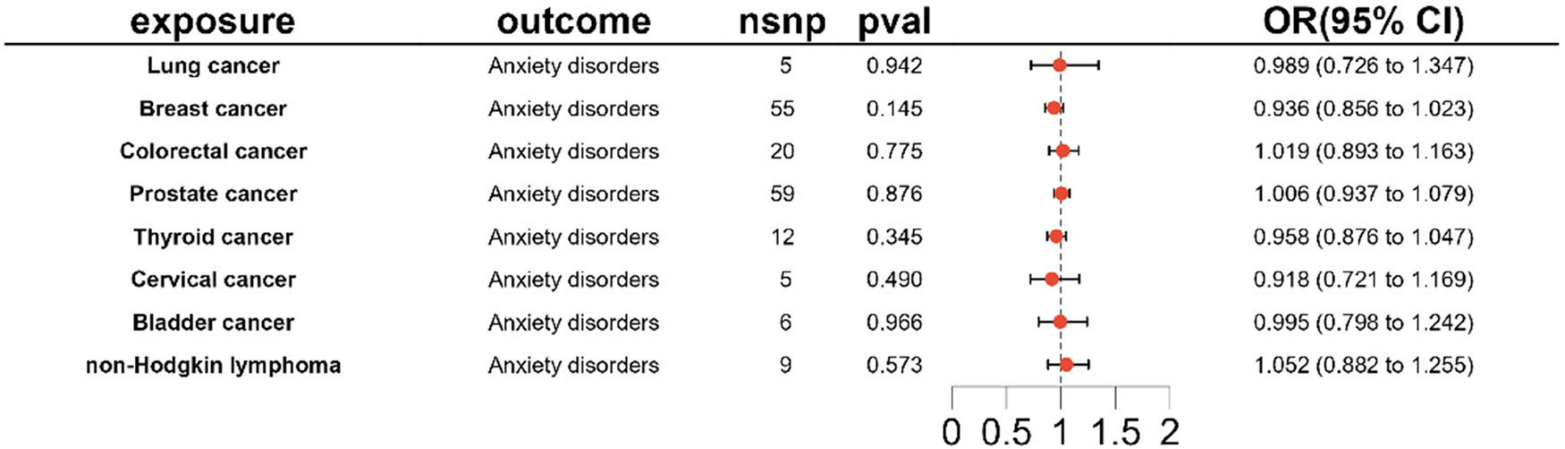
IVW results from a reverse Mendelian study with common cancers as an exposure factor and anxiety disorders as an outcome.

1. Forward MR analysis: IVW analysis, our primary method, revealed no causal association between anxiety disorder and lung cancer (OR 1.013, 95% CI [0.905–1.133], *p* = 0.822). Similarly, no causal relationships were observed between anxiety disorder and other common cancers (*p *> 0.05), as described in Table 1 and Figure 2.
2. Reverse MR analysis: Regrettably, our reverse MR study did not furnish evidence supporting a causal relationship between ten common cancers and anxiety disorders, as depicted in Table 1 and Figure 3.

### 3.2 Sensitivity Analysis

We first assessed heterogeneity in IVW and MR‐Egger regression analyses using the Cochran's *Q* test and its corresponding *p* value. In forward MR analysis, most *p* values were > 0.05, indicating no significant heterogeneity and reinforcing the reliability of our positive results. However, the heterogeneity test *p* value for anxiety disorder with lung cancer and prostate cancer outcomes was < 0.05, suggesting potential variability in effect estimates for these specific analyses. In reverse MR analysis, no significant heterogeneity was observed in IVW or MR‐Egger results. To address this heterogeneity, we performed IVW analysis with a random‐effects model to ensure a robust synthesis of results. Notably, all results were rigorously checked to reduce horizontal pleiotropy and minimize bias, as detailed in Table 2. Additionally, we generated leave‐one‐out and funnel plots to assess the risk of outcome bias. A relatively symmetrical funnel plot indicates a lower risk of outcome bias. Through sensitivity analysis using the SNP exclusion method in leave‐one‐out plots, we found no single SNP significantly affecting the MR results, confirming the robustness of our findings. Scatter plots were also inspected, where each point represents an instrumental variable. Each horizontal solid line in the forest plot represents a single SNP, estimated using the Wald ratio method. All leave‐one‐out plots, scatter plots, funnel plots, and forest plots are available in Figures S1–S18.

## 4 Discussion

Anxiety disorders often present early in somatic diseases and are associated with multiple cancers (Cosci et al. 2015). For instance, anxiety may be an early manifestation of lung cancer, meningioma, and pancreatic cancer (Montazeri et al. 1998; Assefa et al. 2012; Kant 1946). Moreover, severe anxiety can also affect functional outcomes in several cancers, which is particularly evident in male urologic cancers, including prostate and testicular cancers (Dinesh et al. 2021), but has received little attention. A meta‐analysis of 165 studies found that stress‐related psychosocial factors were correlated with increased cancer incidence in initially healthy populations (*p* = *0.005*) (Chida et al. 2008). Another meta‐analysis involving 51 cohort studies and over 2.6 million participants demonstrated significant associations between anxiety/depression and cancer risk (risk ratio [RR] 1.13, 95% CI [1.06–1.19]), cancer‐specific mortality (RR 1.21, 95% CI [1.16–1.26]), and all‐cause mortality (RR 1.24, 95% CI [1.13–1.35]) (Wang et al. 2020). Consistently, anxiety is linked to higher cancer incidence, poorer survival, and elevated cancer‐specific mortality. Anxiety disorders are common in cancer patients and negatively affect both the cost and quality of survival (Erim et al. 2020). Psychiatric issues, including anxiety disorders, are frequent yet overlooked complications in cancer care, impacting quality of life, treatment adherence, survival, and healthcare expenditures (Stark and House 2000; Pitman et al. 2018). Approximately 10% of cancer patients experience anxiety regardless of disease stage (curative treatment or palliative care) (Mitchell et al. 2011). Furthermore, anxiety disorders increase suicide risk across all cancer patient populations (Robson et al. 2010), highlighting a critical concern. However, an individual participant data (IPD) meta‐analysis from the Psychosocial Factors and Cancer Incidence (PSY‐CA) Consortium found no significant associations between anxiety disorders and breast, prostate, colorectal, or alcohol‐related cancers (van Tuijl et al. 2023). Notably, only anxiety symptoms and diagnoses were correlated with the incidence of lung and smoking‐related cancers (hazard ratio [HR], 1.06–1.60) (van Tuijl et al. 2023).

This is the first MR study from our group to investigate the causal relationship between anxiety disorders and common cancers using a genetic approach. The results indicate no causal relationship between anxiety disorders and an increased risk of common cancers. Importantly, no evidence was found to support a causal relationship where common cancers increase the incidence of anxiety disorders. Therefore, a diagnosis of common cancers does not appear to increase the likelihood of developing anxiety disorders. While it is well‐established that many cancer patients experience psychiatric disorders like anxiety and depression, these conditions are more likely driven by environmental factors and personal circumstances than genetic predisposition.

The lexicon surrounding cancer is often perceived as socially menacing and has been identified as a trigger for anxiety (Mogg et al. 1995). Various factors contribute to the prevalence of anxiety disorders in cancer patients, which we summarize into three categories: patient demographics (age, gender, economic status), disease‐related characteristics (pain, cancer type, stage, treatment modality, and severity of complications), and psychosocial factors (family, social, and healthcare support) (Yan et al. 2019). Regarding patient demographics, studies indicate that anxiety is more prevalent among younger cancer patients (Zeilinger et al. 2022). For instance, a 5‐year UK longitudinal study found that younger age was a significant risk factor for anxiety in women following a breast cancer diagnosis (C. Burgess et al. 2005). Younger patients may experience greater anxiety due to limited experience and preparedness in managing physical changes, as well as emotional and cognitive difficulties in accepting their diagnosis (Zeilinger et al. 2022). This group also faces challenges related to body image, self‐perception, and existential concerns, strongly associated with anxiety during the breast cancer survivorship phase (Shi et al. 2022; Morales‐Sánchez et al. 2021). Furthermore, younger patients frequently experience major disruptions to their daily lives after a cancer diagnosis (Linden et al. 2012). Conversely, female patients are prone to anxiety symptoms, likely due to greater willingness to express symptoms and a preference for emotional coping strategies (Salm et al. 2021). Economic factors also play a key role: 43% of thyroid cancer survivors reported financial hardship, which was associated with increased anxiety, reduced productivity, difficulties in social integration, and a vicious cycle (Mongelli et al. 2020).

Cancer type, stage, treatment modality, and cancer‐ or treatment‐related symptoms are key cancer‐specific factors associated with anxiety (Shi et al. 2022; Ikhile et al. 2024). Specifically, patients with aggressive cancer types, advanced disease stages, or severe cancer‐ or treatment‐related symptoms report higher anxiety levels (Cheng et al. 2022). The findings indicate a close association between patients' hospitalization due to sudden deterioration in health and anxiety, exacerbated by symptoms requiring urgent intervention such as uncontrolled pain or shortness of breath, which can impact mental well‐being (Bryniarski et al. 2022). The authors of one study noted that the onset of anxiety disorders and emotional maladjustment was associated with more intense pain produced in the abdominal cavity (Niles and O'Donovan 2019; Zvolensky et al. 2018). Anxiety incidence in advanced cancer patients is also associated with significant weight loss (> 10%), elevated performance status scores, treatment type, and prolonged treatment duration (Park et al. 2019; Park et al. 2018). Surgical interventions for cancer are generally well‐received by patients, despite high levels of preoperative anxiety regarding the success of the procedure. Post‐surgery, this anxiety typically subsides, indicating that surgical anxiety is typically short‐lived (Stark and House 2000). Patients who undergo mastectomy report higher anxiety scores 5–6 years post‐diagnosis than those who receive breast‐conserving surgery, likely due to body image distress and chronic pain (Breidenbach et al. 2022; Unukovych et al. 2012; Nishimura et al. 2017), as previously noted. Chemotherapy and radiotherapy also trigger anxiety, though context‐dependent. After adjusting for disease progression and physical status, chemotherapy‐induced toxicity is strongly correlated with anxiety, suggesting patients perceive toxicity as a threat when it occurs (Schag and Heinrich 1989). For instance, 70%–80% of patients with non‐muscle invasive bladder cancer require cystectomy plus bladder infusion chemotherapy (Bhanvadia 2018), and the prolonged infusion duration increases anxiety levels (Vartolomei et al. 2020). Some patients lose confidence in treatment, delaying or discontinuing chemotherapy and compromising overall well‐being. Reduced quality of life further erodes self‐esteem and exacerbates anxiety. However, anxiety is most severe in patients with inoperable tumors who have no access to radiotherapy or other therapeutic options, likely because the lack of intervention signals inevitable health decline (Hughes 1987). Frequent follow‐up visits also contribute to anxiety. For example, prostate cancer patients report heightened anxiety before regular prostate‐specific antigen (PSA) tests, with nearly one‐third experiencing pretest anxiety (Meissner et al. 2023; Dale et al. 2005). Conversely, anxiety levels tended to diminish over time after cancer recurrence, likely due to adaptation to the chronic disease trajectory (Corbin 1998; Reed and Corner 2015).

Our study offers several key strengths. First, MR analysis by simulating genetic randomized controlled trials within an observational design—yields robust causal evidence. Leveraging randomly inherited SNPs as instrumental variables, MR minimizes confounding biases inherent in observational research and eliminates reverse causality, thereby enhancing the credibility of our causal inferences. Second, our preliminary finding of no causal association between anxiety disorders and cancer substantially reduces the costs and time associated with traditional clinical trials. This approach facilitates preclinical research advancement while adhering to ethical guidelines. Our results highlight the need for expanded cancer screening in individuals with a genetic predisposition to anxiety disorders and underscore the necessity of addressing individual‐disease‐social factor interactions post‐diagnosis to lower anxiety disorder incidence. However, our study has limitations. First, the GWAS data used in our analysis are exclusively from European populations, necessitating further research to validate findings in diverse racial/ethnic groups and mitigate potential population biases. Second, the number of SNPs meeting our inclusion criteria for anxiety disorders and specific cancer types is relatively small, introducing potential bias. The heterogeneity of the cancer patient population underscores the complexity of anxiety disorders as a potential pathogenic factor. Future studies should explore multiple cancer subtypes in larger cohorts to more comprehensively elucidate specific causal relationships.

## 5 Conclusion

Our inaugural two‐way MR study investigated the relationship between anxiety disorders and prevalent cancers (lung, breast, colorectal, prostate, stomach, liver, thyroid, cervical, bladder, and non‐Hodgkin lymphoma). From a genetic perspective, we found no association between anxiety disorders and common cancer risk. Furthermore, reverse MR analyses did not support a causal link from common cancers to anxiety disorders. The prevalence of anxiety disorders in cancer patients can be attributed to various factors including general conditions (such as age, gender, and economic status), characteristics intrinsic to the disease itself (such as pain, cancer type, stage, treatment modalities, and severity of complications), and psychosocial factors (including family support, social networks, and medical care availability). Moving forward, future research should prioritize larger, more recent datasets. Comprehensive studies covering diverse cancer subtypes are critical to fully investigate and clarify specific causal relationships.

## Author Contributions

Study design and manuscript writing were done by J.S. and J.Z. Literature search and data collection were done by J.W. and J.S. Figures, tables, and data analysis were done by J.W. and J.Z. Manuscript revision and article polish were done by J.S. and J.Z. Manuscript finalization and funds support were done by J.W. All authors read and approved the final manuscript.

## Ethics Statement

The authors have nothing to report.

## Conflicts of Interest

The authors declare no conflicts of interest.

## Peer Review

The peer review history for this article is available at https://publons.com/publon/10.1002/brb3.71002

## Supporting information




**Supplementary Material**: brb371002‐sup‐0001‐SuppMat.docx

## Data Availability

Data used in this study are available in the supplementary materials. The dataset presented herein can be accessed via an online database, whose name is provided in the Supplementary material. The original contributions presented in the study are included in the article/Supplementary material, further inquiries can be directed to the corresponding authors.
