# Supplementary material for: Causal Relationship Between Anxiety Disorders and Cancers: A Bidirectional Two‐Sample Mendelian Randomization Study
Source: Brain Behav. 2025 Oct 15;15(10):e71002. doi: 10.1002/brb3.71002 (PMC12528808; doi:10.1002/brb3.71002)
Supplement: Supplementary file 1 — Supplementary Material: brb371002‐sup‐0001‐SuppMat.docx [file BRB3-15-e71002-s001.docx]

**
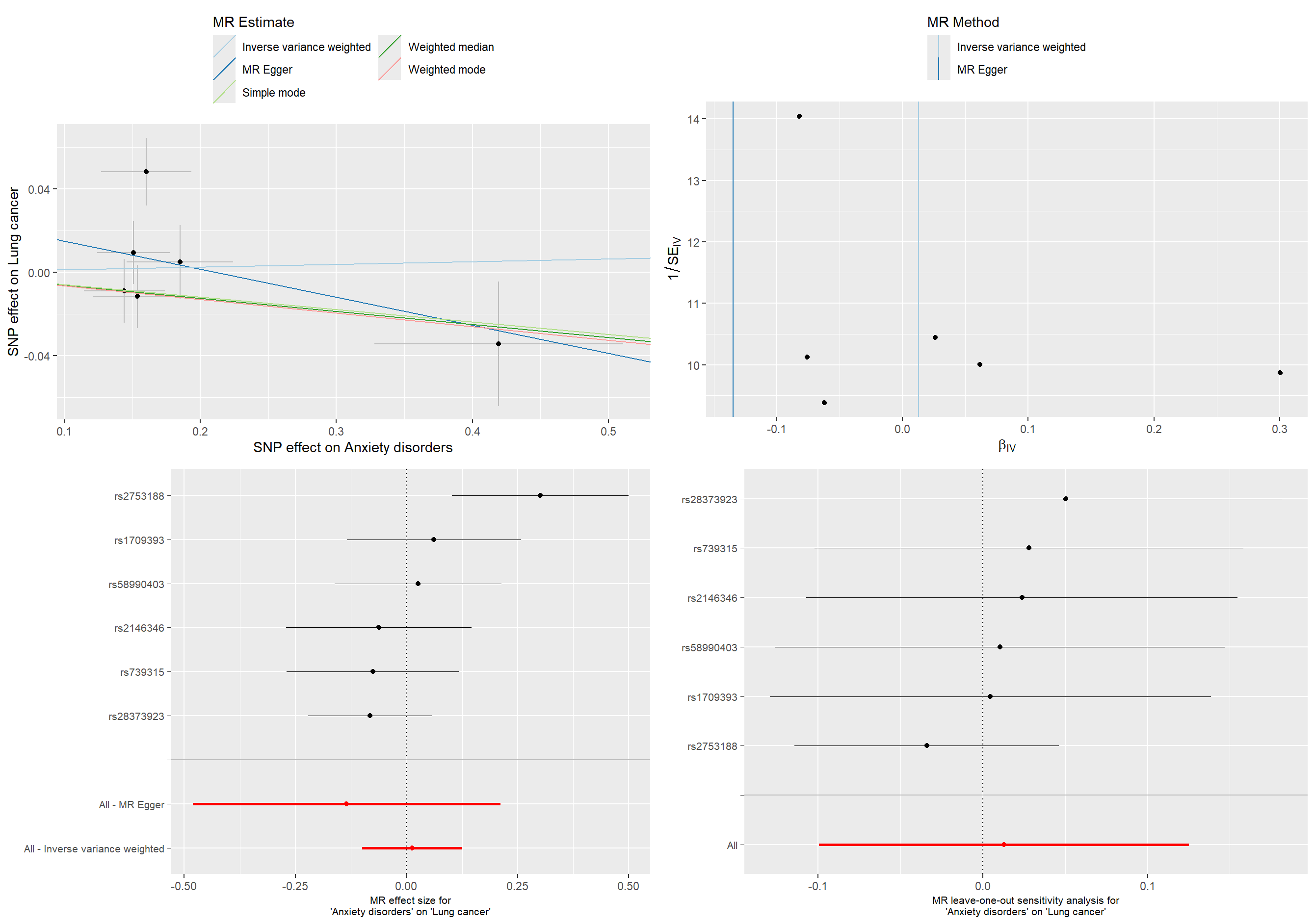
**

**Figure S1: The causal effect of** **Anxiety disorders on Lung cancer. This diagram includes scatter plots, funnel plots, forest plots and leave one method plots.**

**
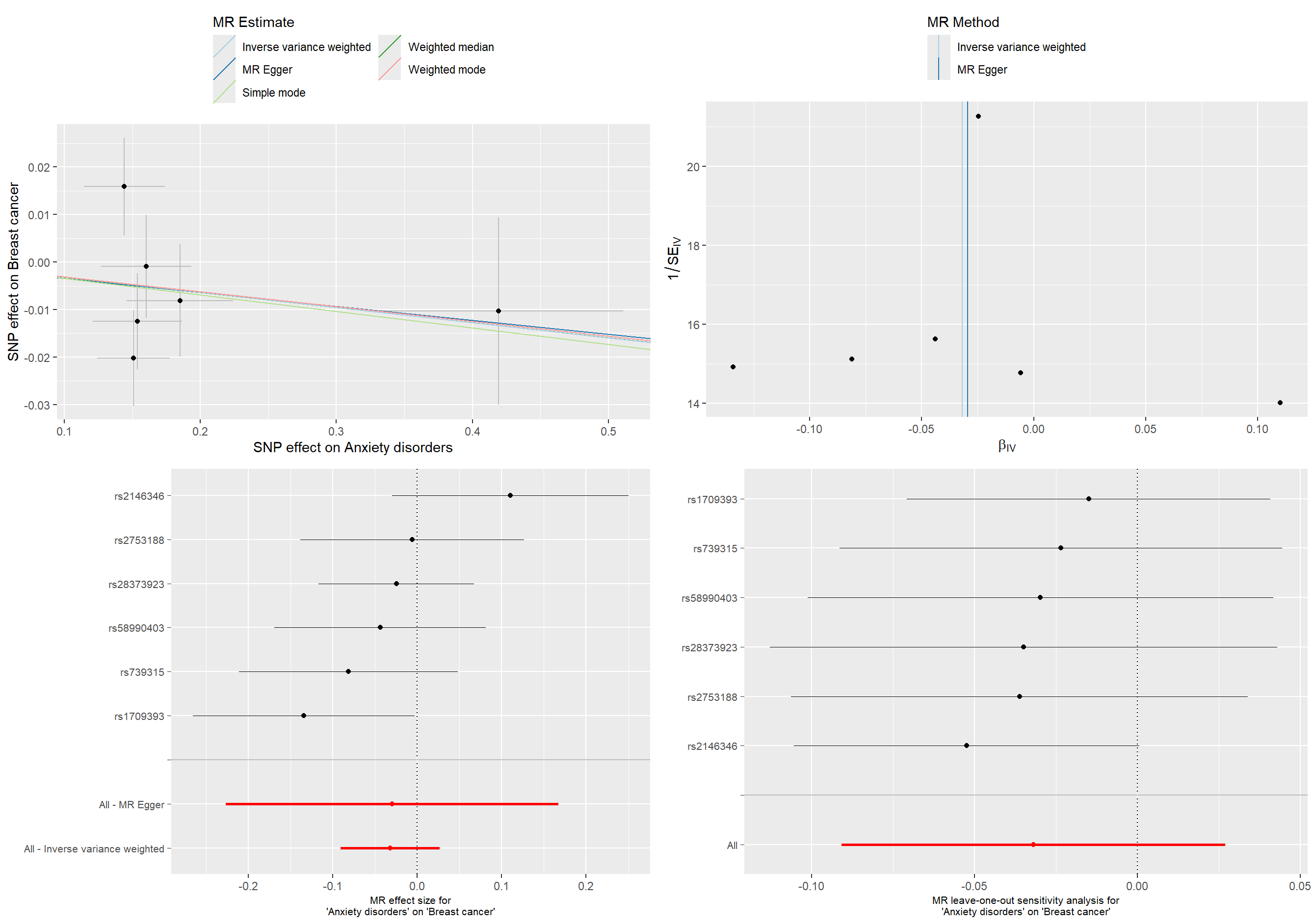
**

**Figure S2: The causal effect of** **Anxiety disorders on Breast cancer. This diagram includes scatter plots, funnel plots, forest plots and leave one method plots.**

**
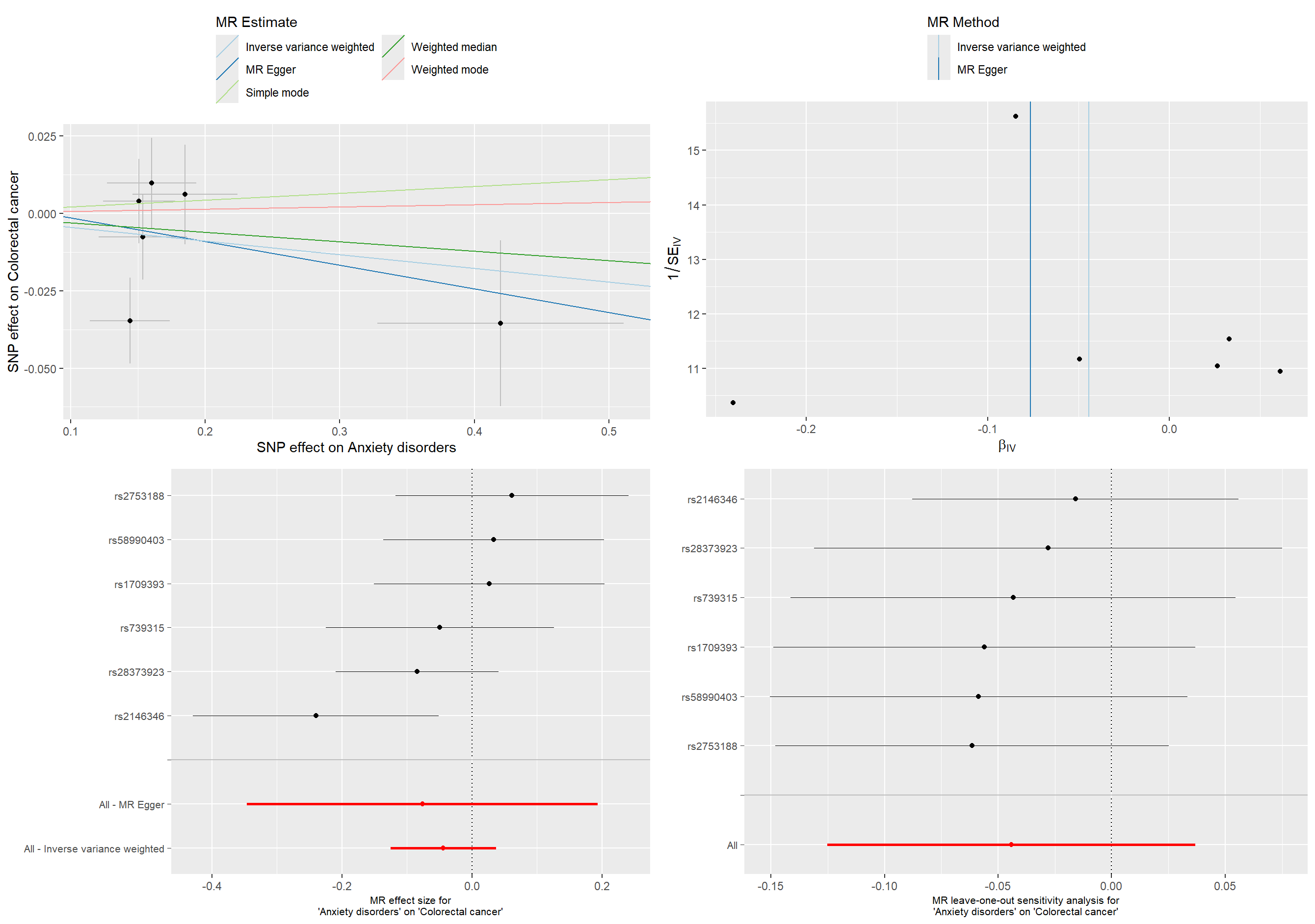
**

**Figure S3: The causal effect of** **Anxiety disorders on Colorectal cancer. This diagram includes scatter plots, funnel plots, forest plots and leave one method plots.**

**
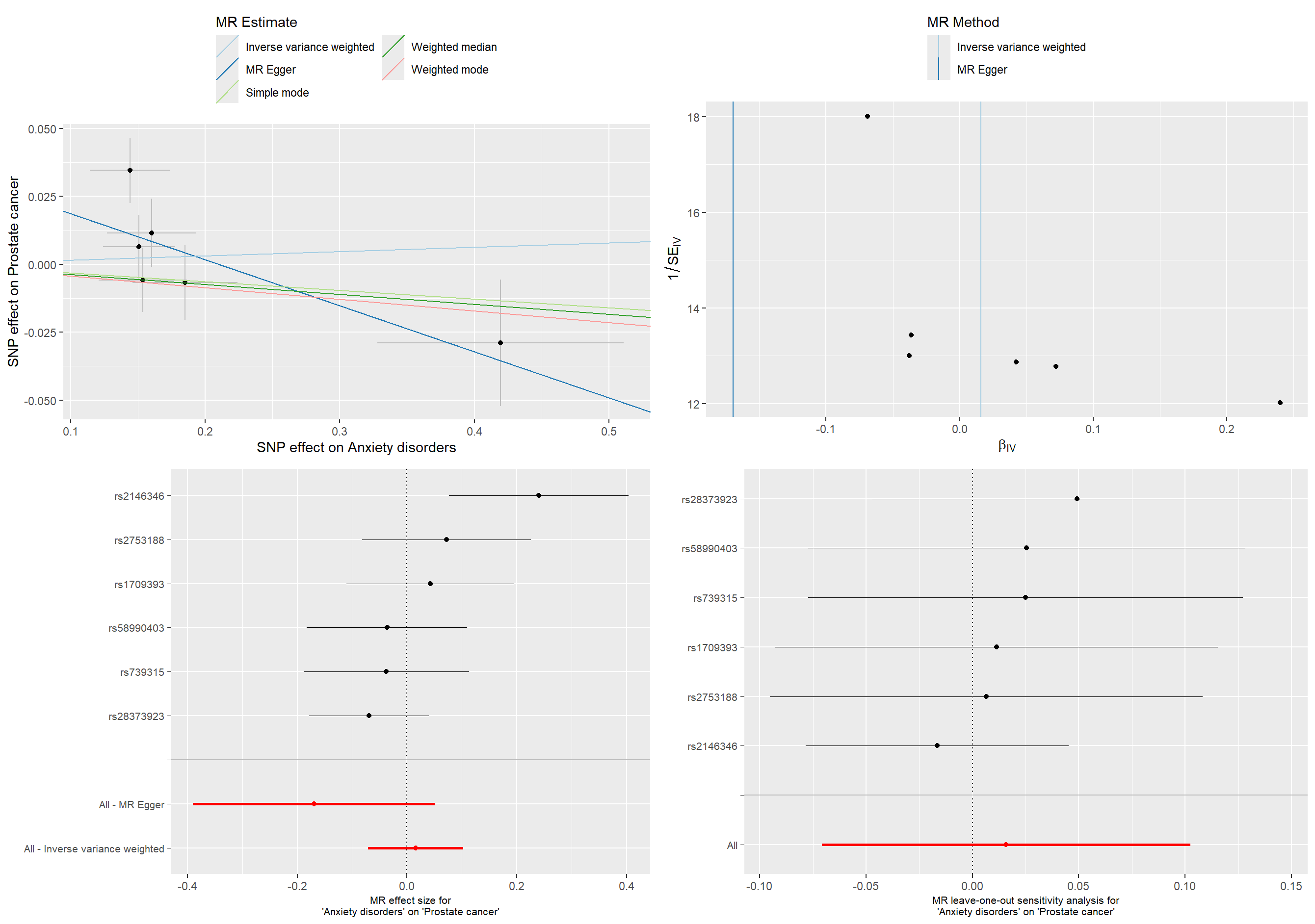
**

**Figure S4: The causal effect of** **Anxiety disorders on Prostate cancer. This diagram includes scatter plots, funnel plots, forest plots and leave one method plots.**

**
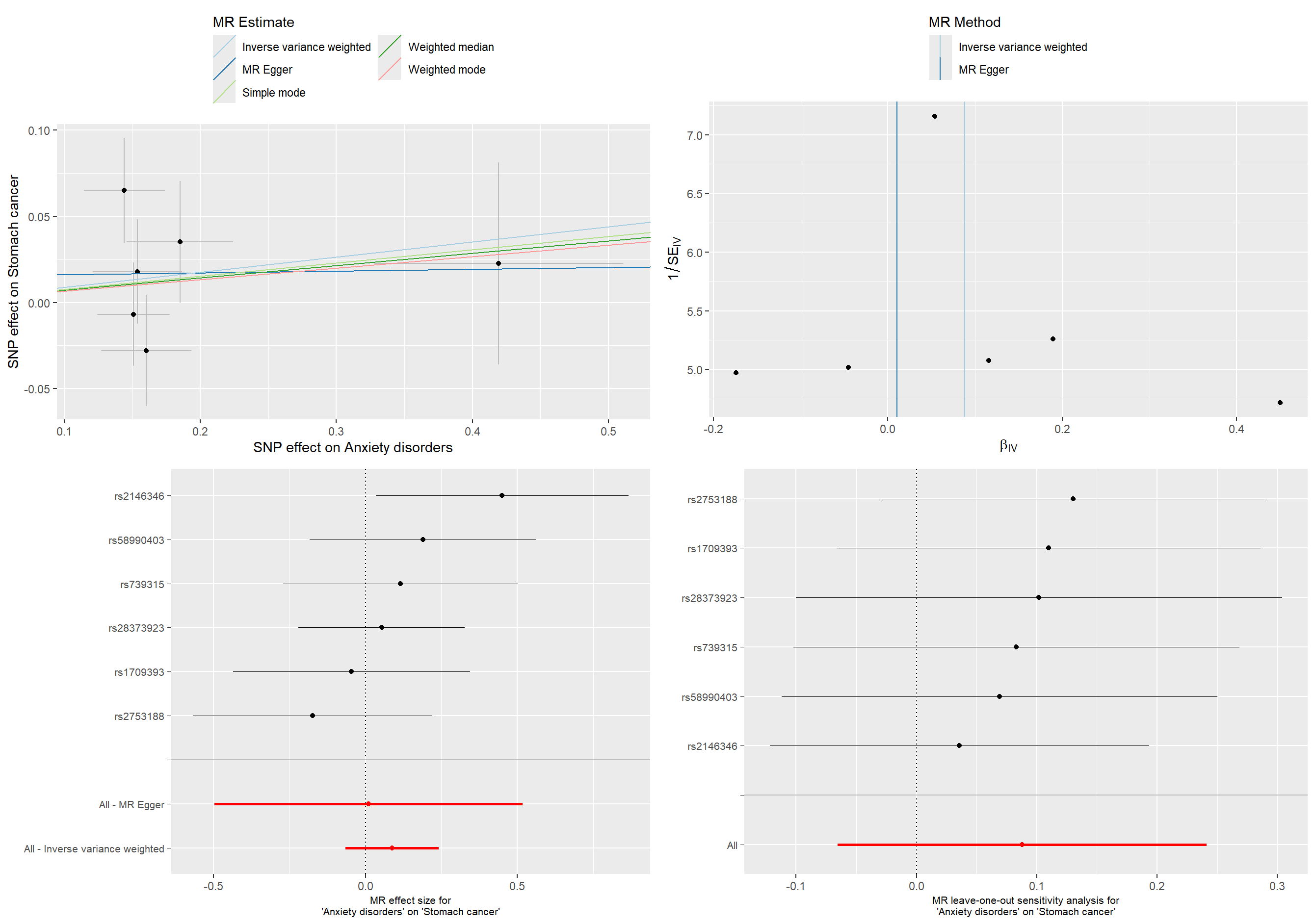
**

**Figure S5: The causal effect of** **Anxiety disorders on Stomach cancer. This diagram includes scatter plots, funnel plots, forest plots and leave one method plots.**


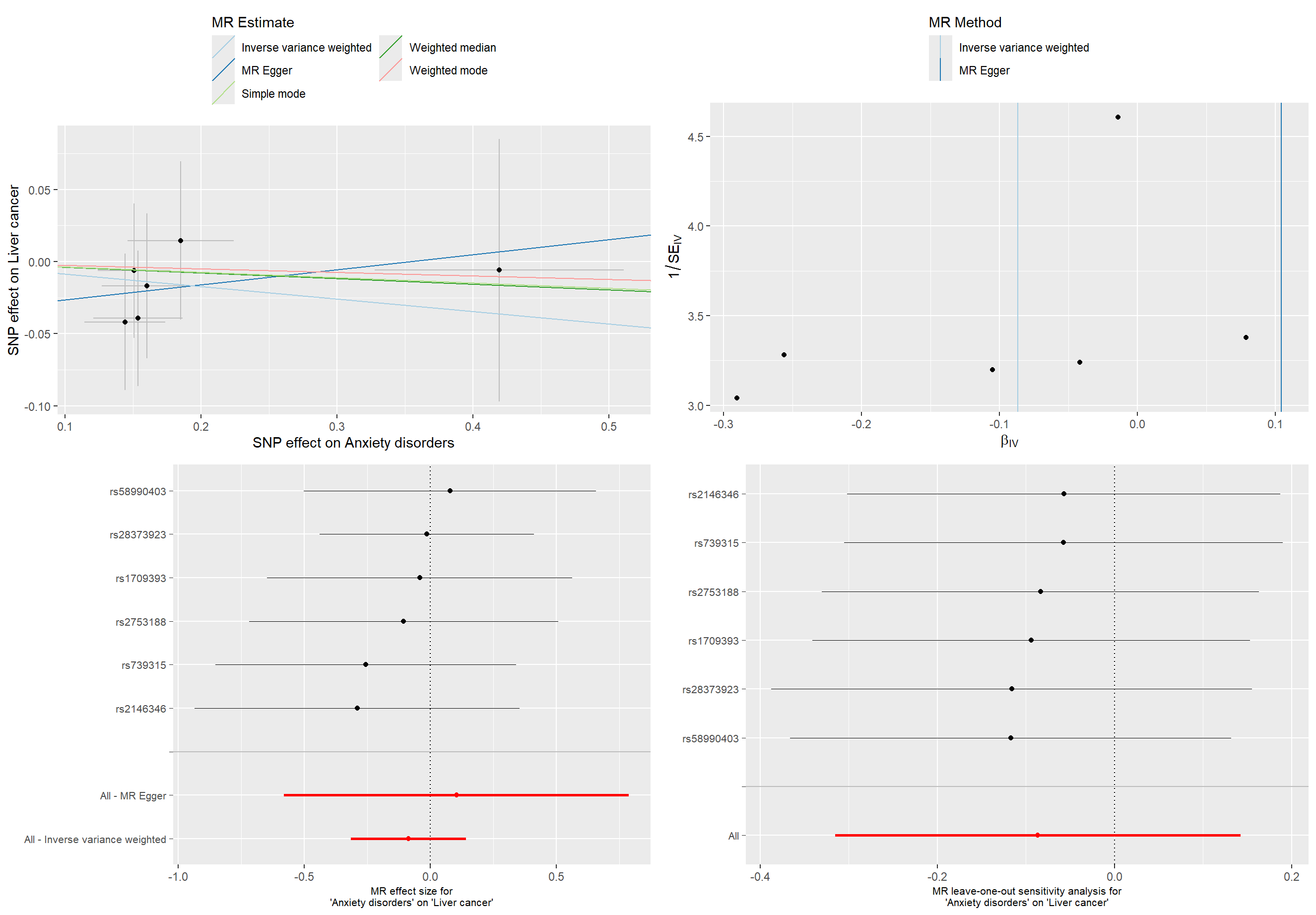


**Figure S6: The causal effect of** **Anxiety disorders on Liver cancer. This diagram includes scatter plots, funnel plots, forest plots and leave one method plots.**

**
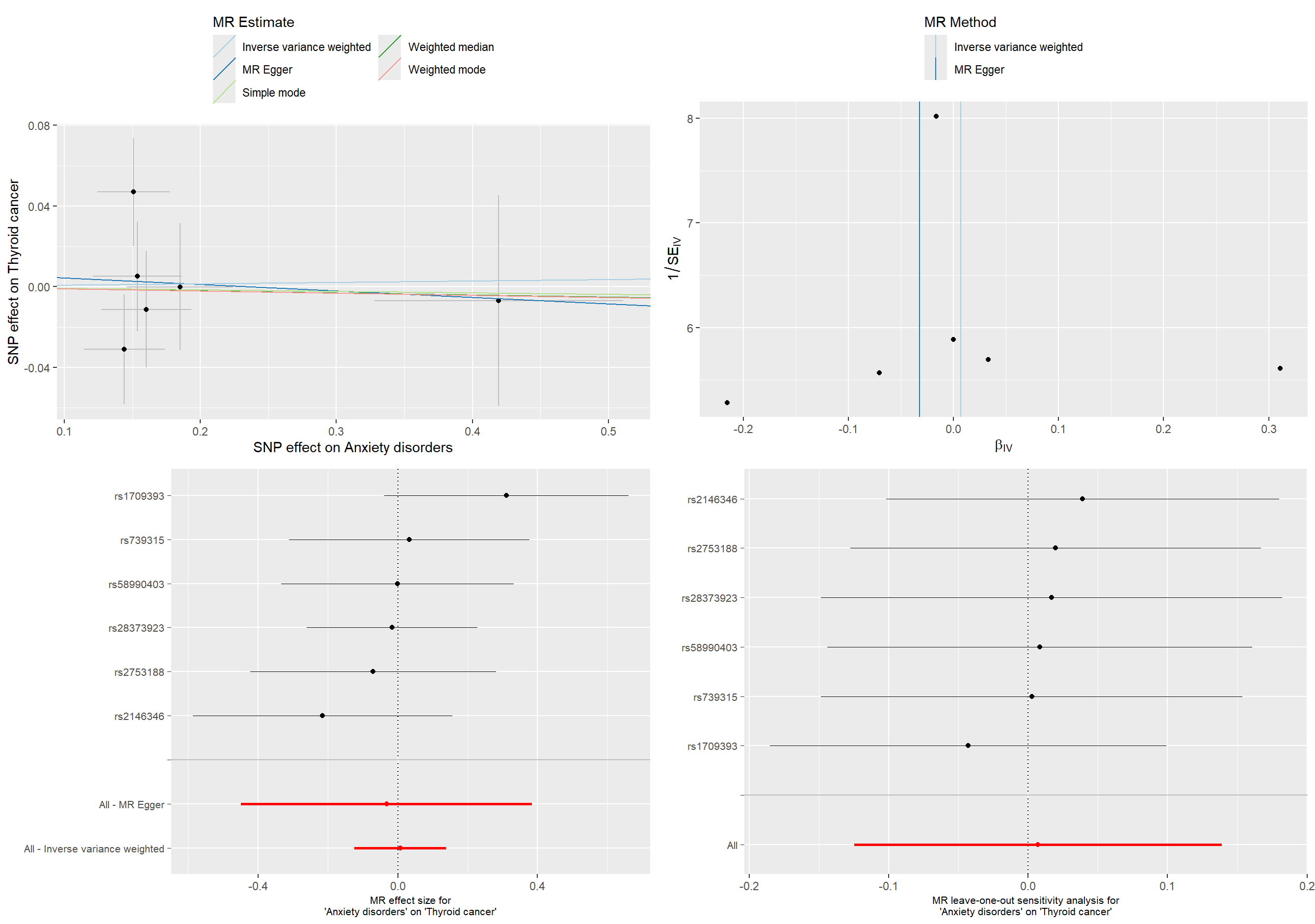
**

**Figure S7: The causal effect of** **Anxiety disorders on Thyroid cancer. This diagram includes scatter plots, funnel plots, forest plots and leave one method plots.**

**
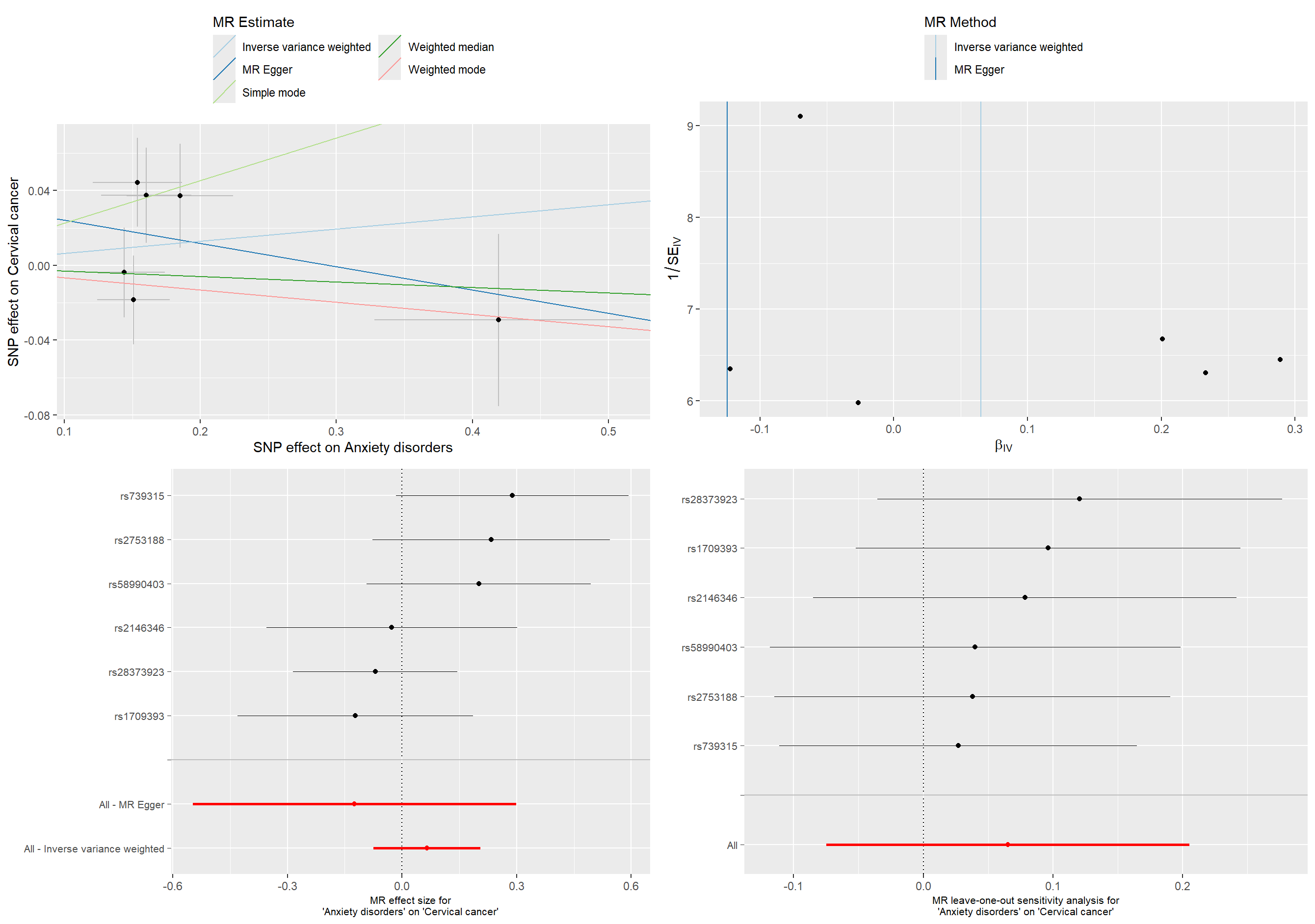
**

**Figure S8: The causal effect of** **Anxiety disorders on Cervical cancer. This diagram includes scatter plots, funnel plots, forest plots and leave one method plots.**

**
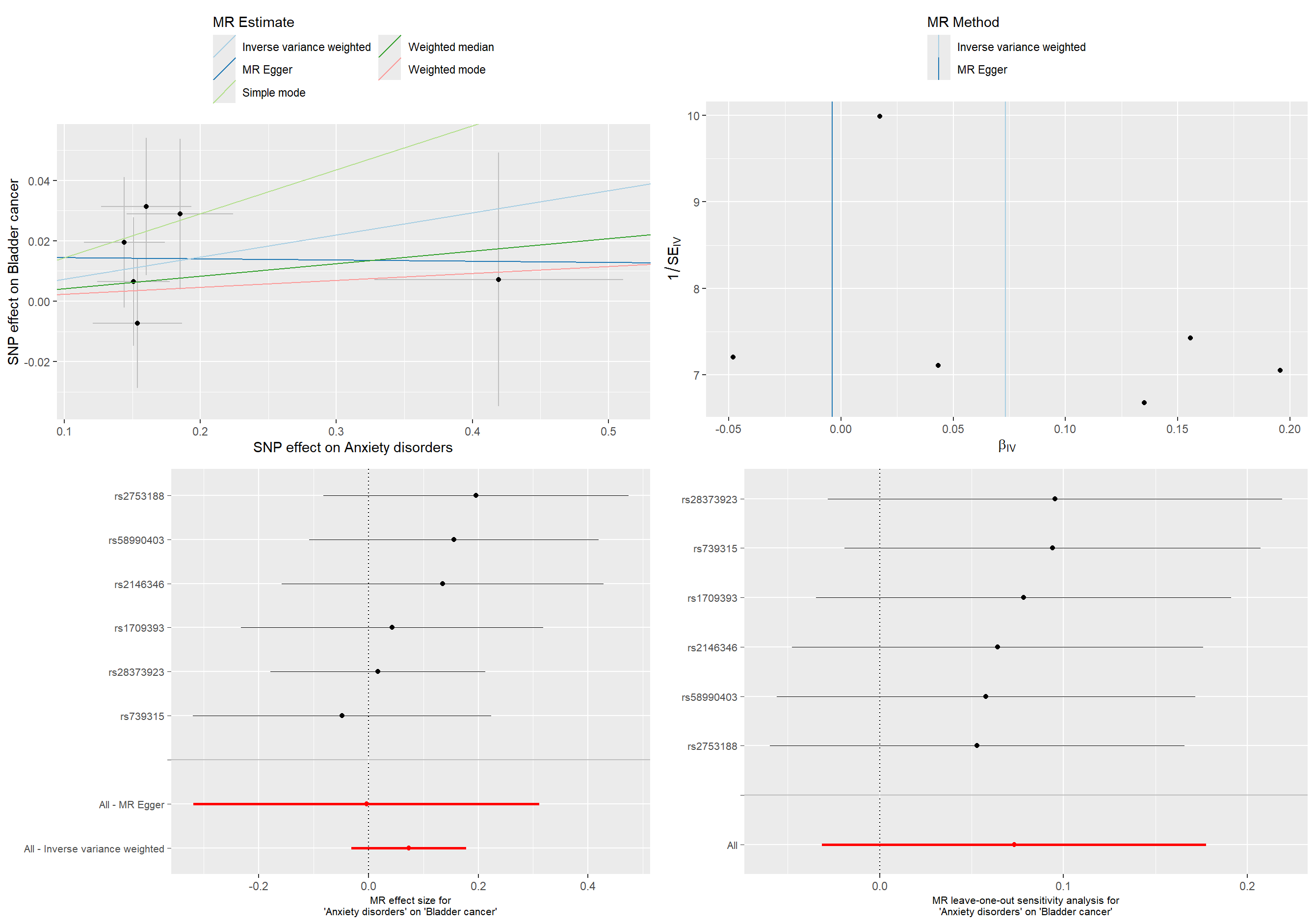
**

**Figure S9: The causal effect of** **Anxiety disorders on Bladder cancer. This diagram includes scatter plots, funnel plots, forest plots and leave one method plots.**


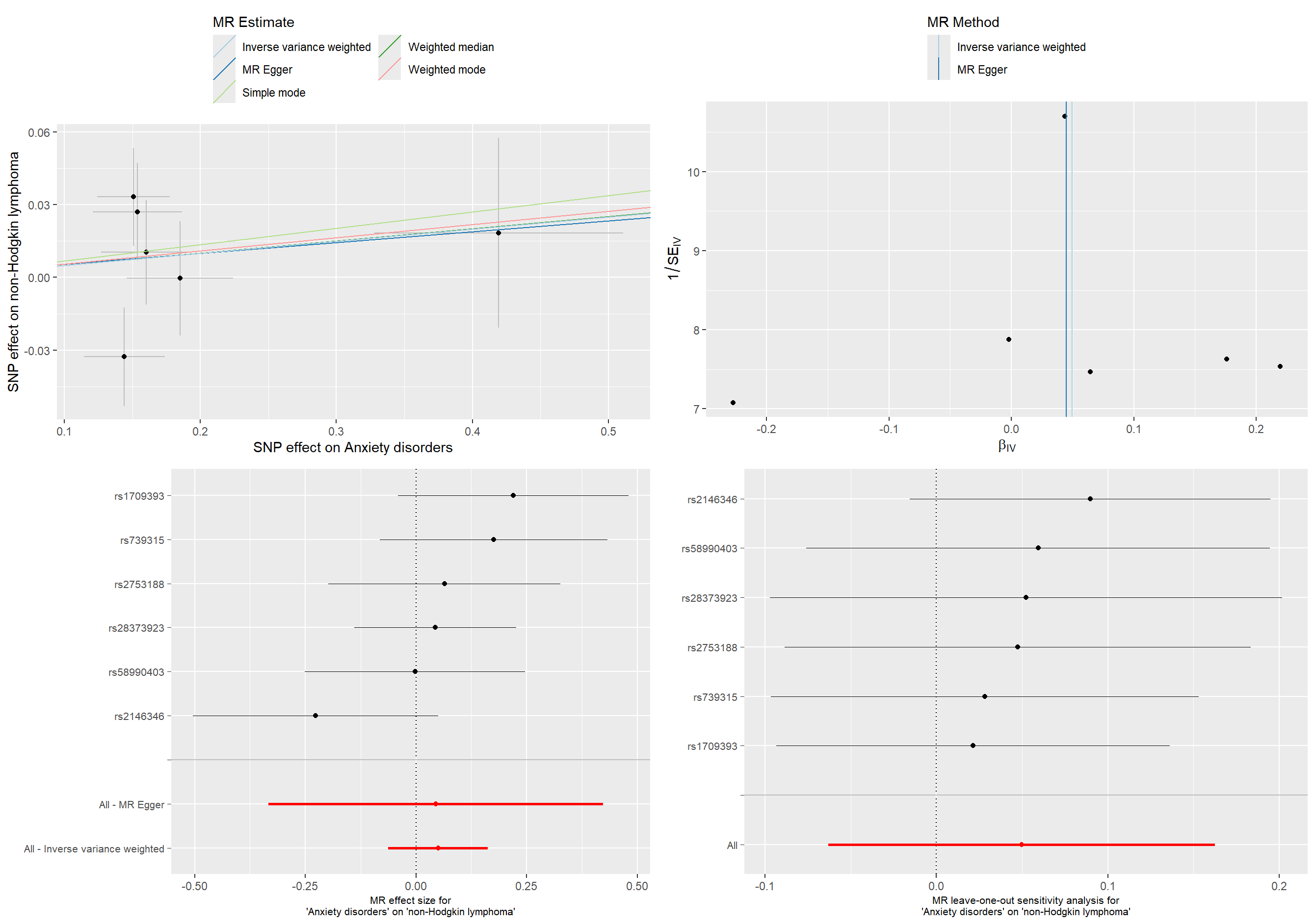


**Figure S10: The causal effect of** **Anxiety disorders on non-Hodgkin lymphoma. This diagram includes scatter plots, funnel plots, forest plots and leave one method plots.**

**
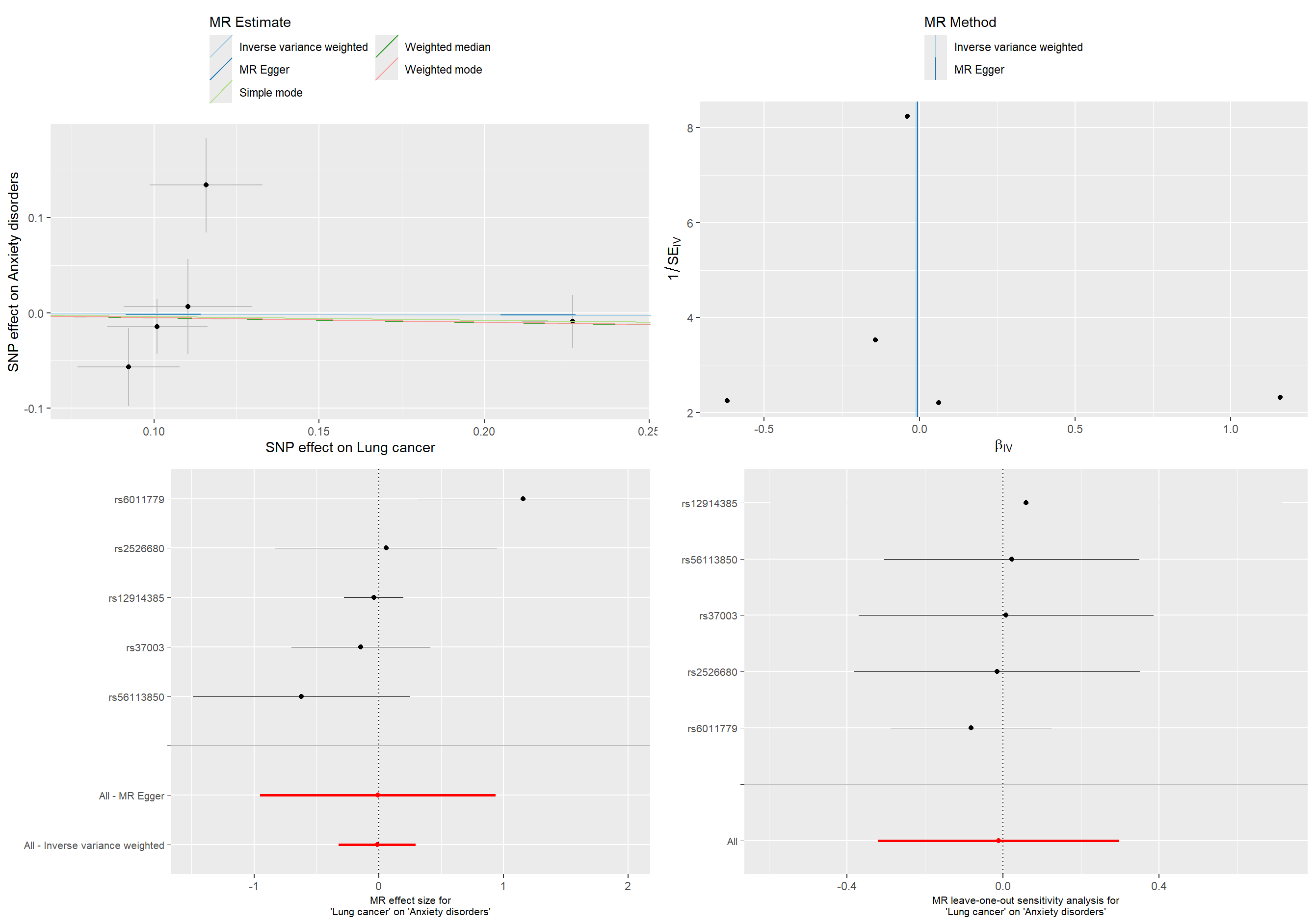
**

**Figure S11: The causal effect of** **Lung cancer on Anxiety disorders. This diagram includes scatter plots, funnel plots, forest plots and leave one method plots.**

**
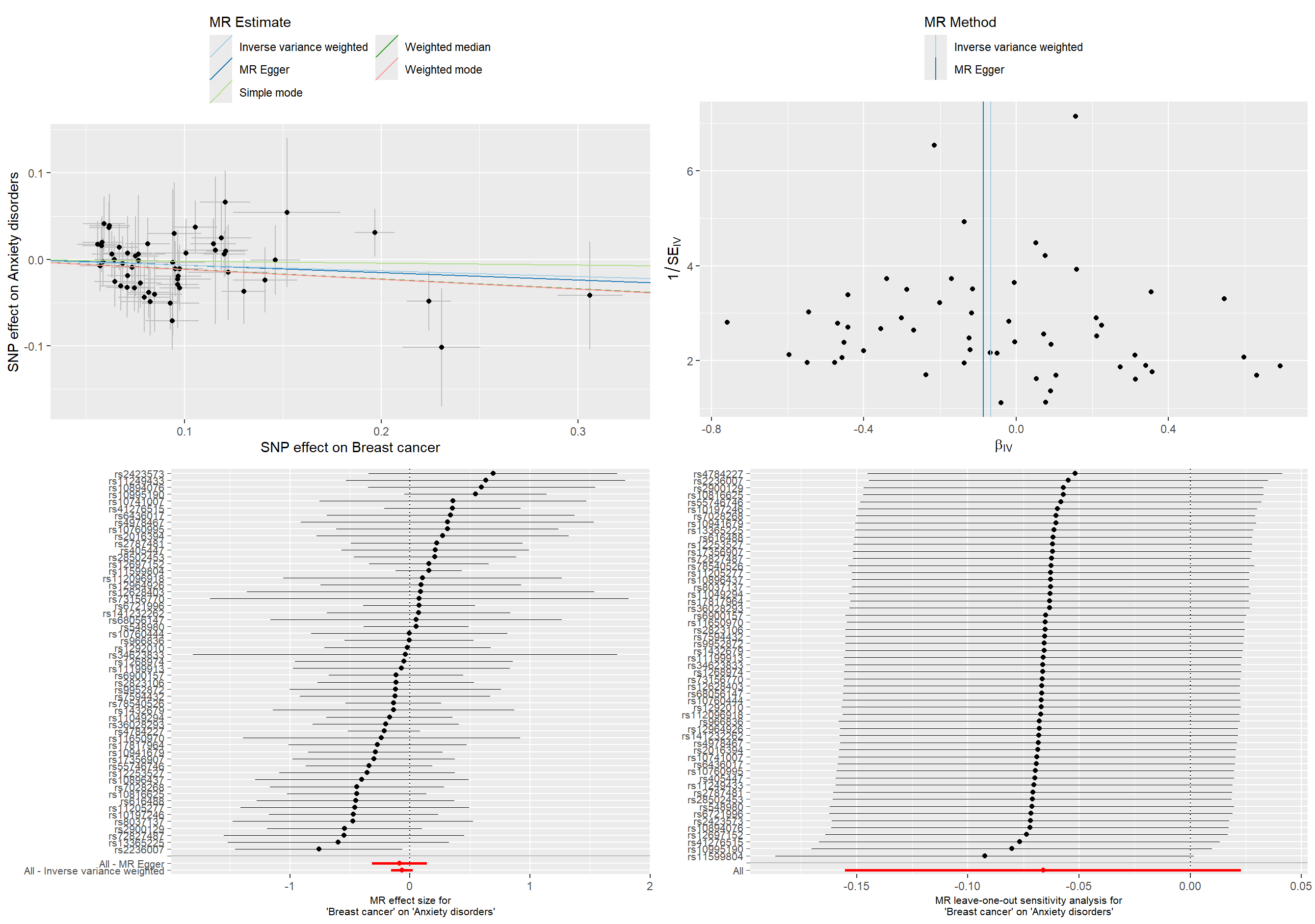
**

**Figure S12: The causal effect of** **Breast cancer on Anxiety disorders. This diagram includes scatter plots, funnel plots, forest plots and leave one method plots.**

**
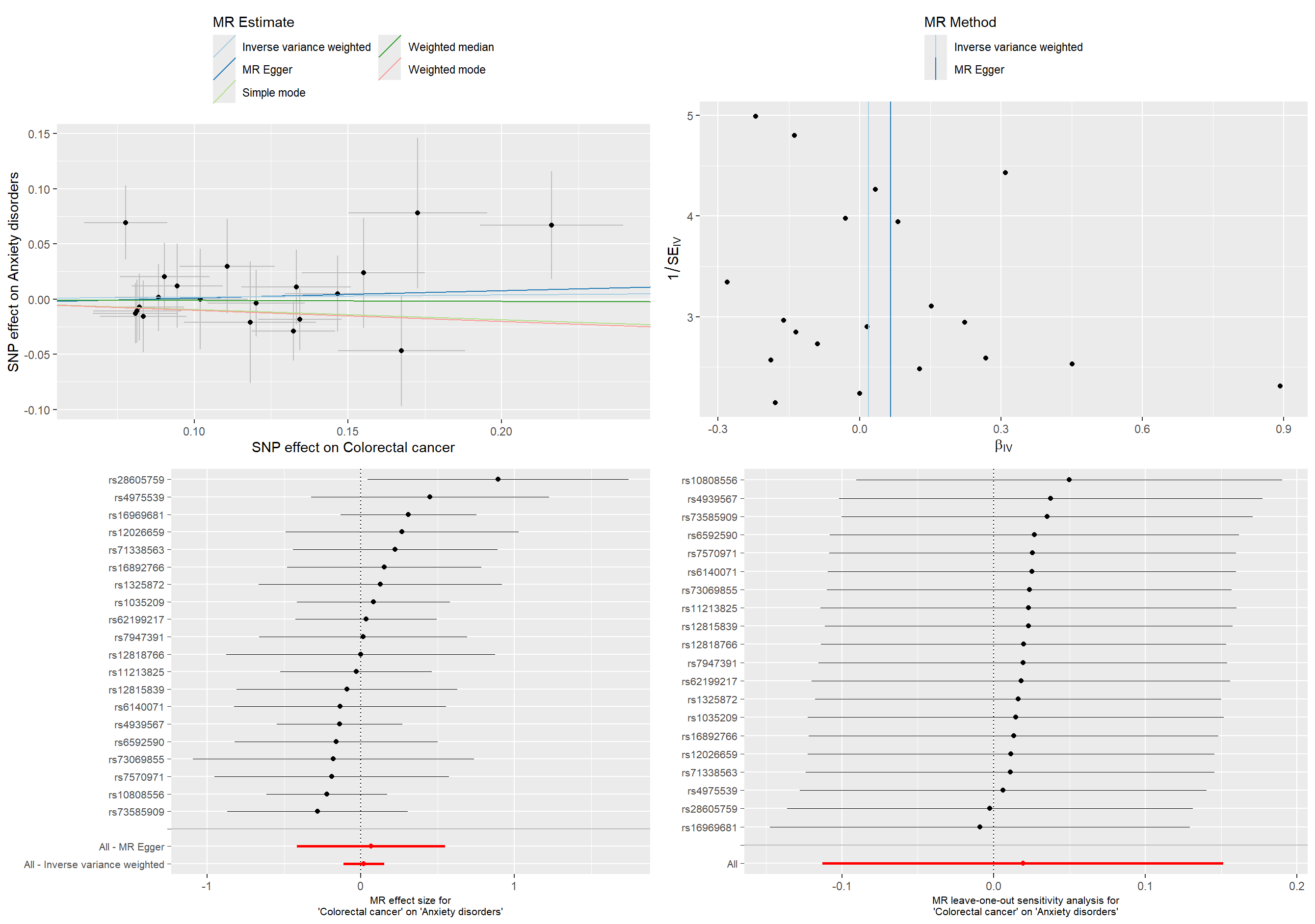
**

**Figure S13: The causal effect of** **Colorectal cancer on Anxiety disorders. This diagram includes scatter plots, funnel plots, forest plots and leave one method plots.**

**
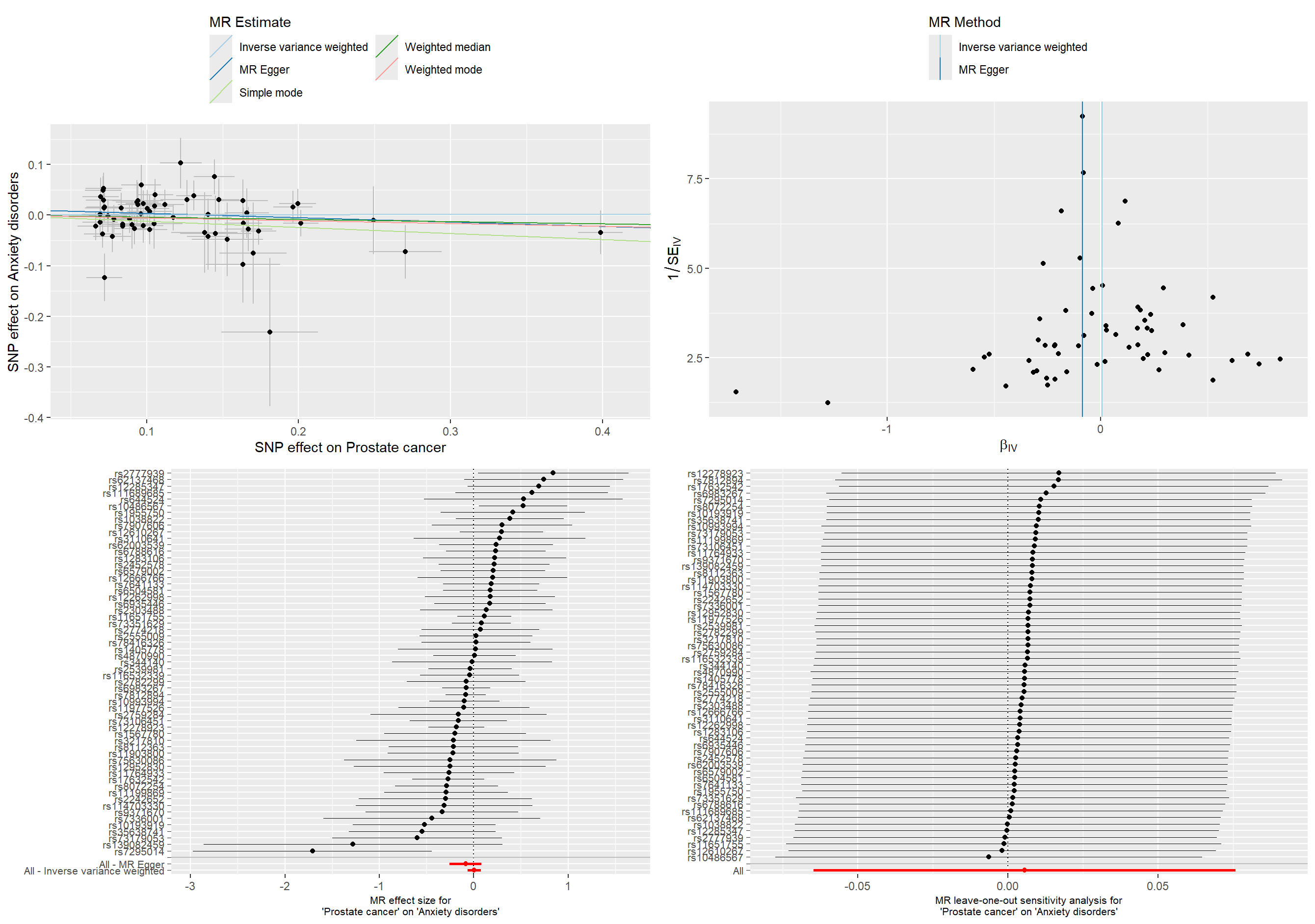
**

**Figure S14: The causal effect of** **Prostate cancer on Anxiety disorders. This diagram includes scatter plots, funnel plots, forest plots and leave one method plots.**

**
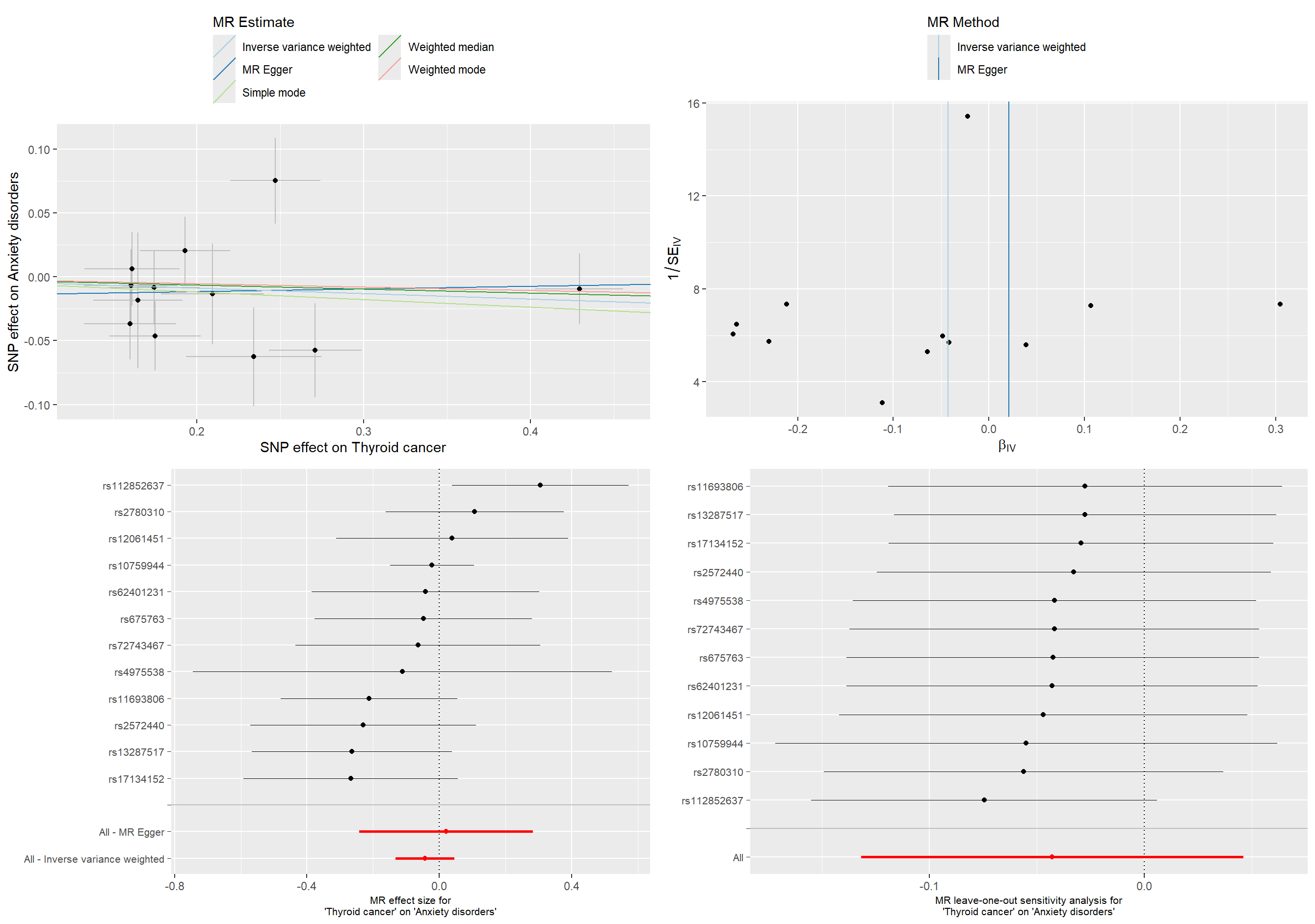
**

**Figure S15: The causal effect of** **Thyroid cancer on Anxiety disorders. This diagram includes scatter plots, funnel plots, forest plots and leave one method plots.**

**
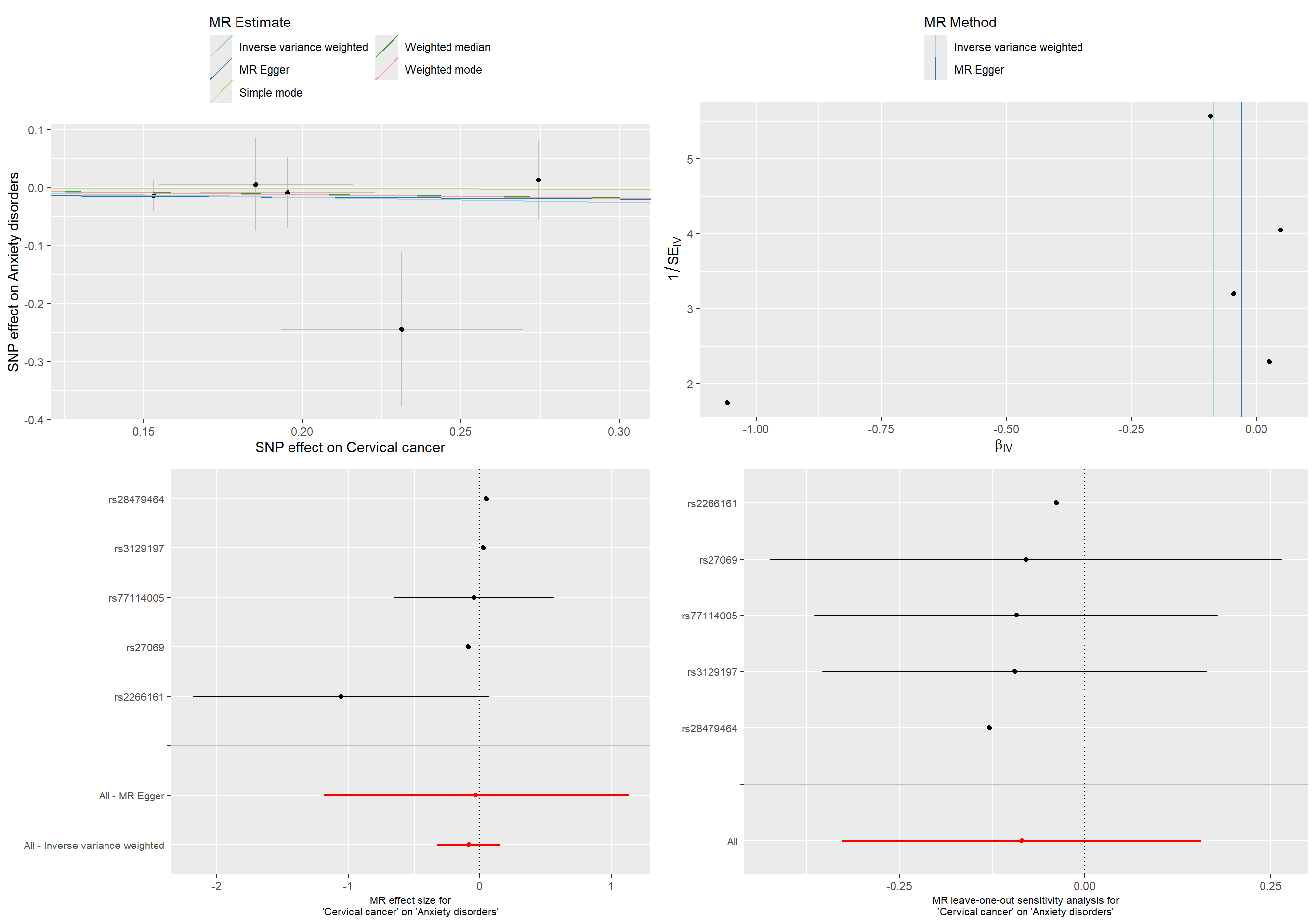
**

**Figure S16: The causal effect of** **Cervical cancer on Anxiety disorders. This diagram includes scatter plots, funnel plots, forest plots and leave one method plots.**

**
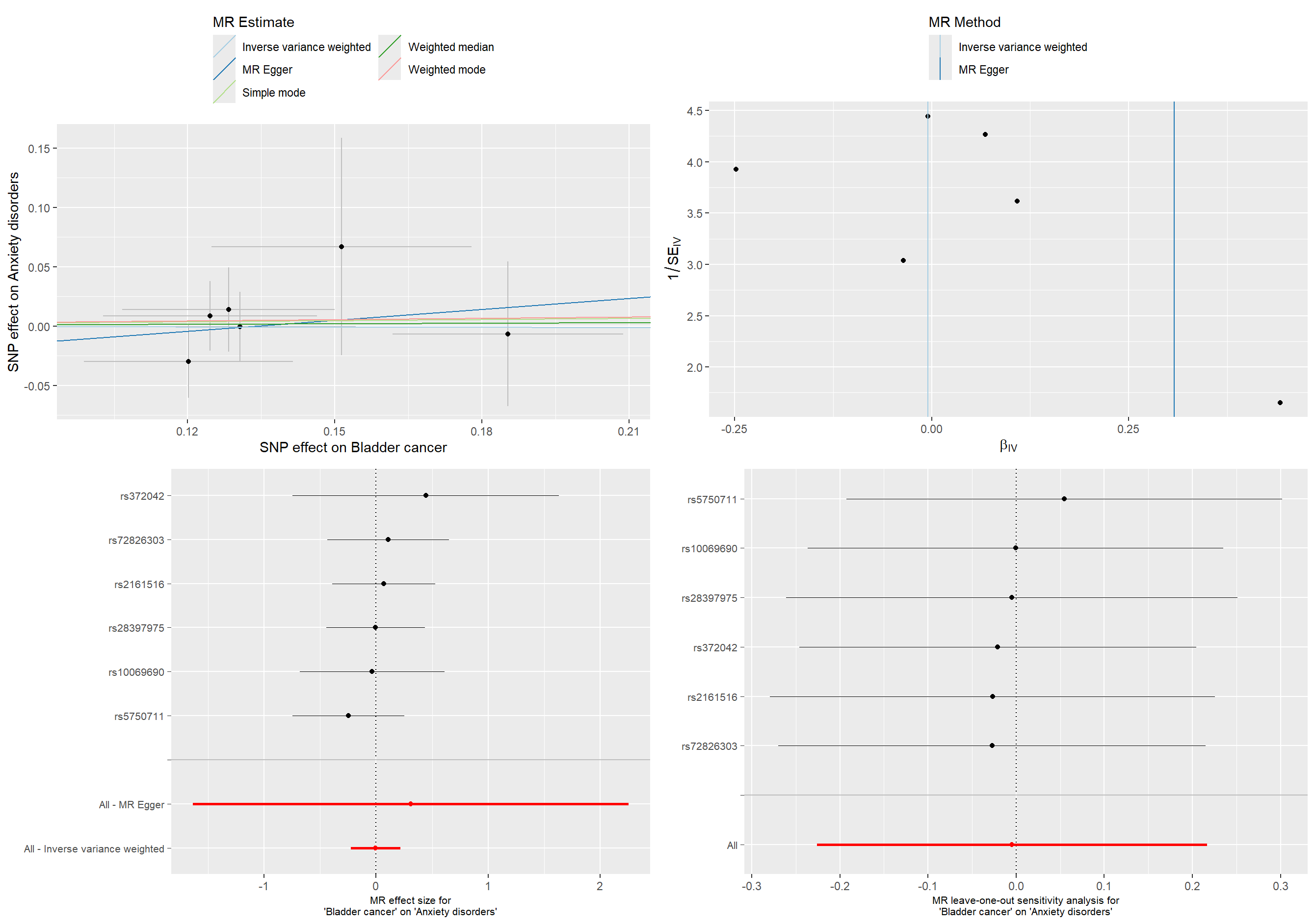
**

**Figure S17: The causal effect of** **Bladder cancer on Anxiety disorders. This diagram includes scatter plots, funnel plots, forest plots and leave one method plots.**

**
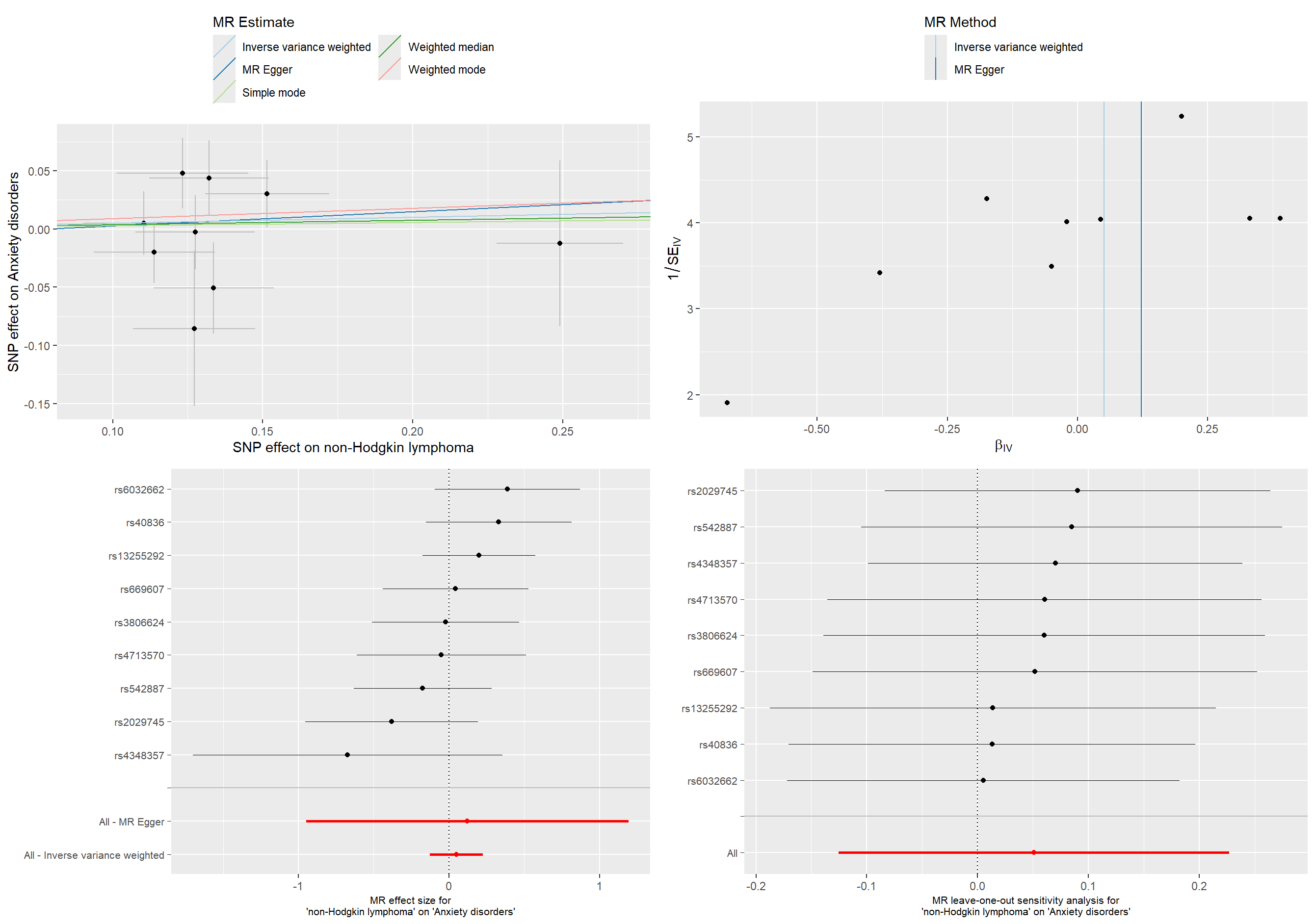
**

**Figure S18: The causal effect of** **non-Hodgkin lymphoma on Anxiety disorders. This diagram includes scatter plots, funnel plots, forest plots and leave one method plots.**

**Supplementary Table 1. Genetic data characteristics of anxiety disorders and common cancers**

| **Trait** | **Source** | **Dataset** | **ncase** | **ncontrol** | **Sample size** |
| --- | --- | --- | --- | --- | --- |
| Anxiety disorders | PGC | / | 5580 | 17310 | 22890 |
| Lung cancer | FinnGen | finngen_R12_C3_BRONCHUS_LUNG_EXALLC | 9639 | 378749 | 388388 |
| Breast cancer | FinnGen | finngen_R12_C3_BREAST_EXALLC | 24270 | 222078 | 246348 |
| Colorectal cancer | FinnGen | finngen_R12_C3_COLORECTAL_EXALLC | 11790 | 378749 | 390539 |
| Prostate cancer | FinnGen | finngen_R12_C3_PROSTATE_EXALLC | 20368 | 156671 | 177039 |
| Stomach cancer | FinnGen | finngen_R12_C3_STOMACH_EXALLC | 2296 | 378749 | 381045 |
| Liver cancer | FinnGen | finngen_R12_C3_HEPATOCELLU_CARC_EXALLC | 947 | 378749 | 379696 |
| Thyroid cancer | FinnGen | finngen_R12_C3_THYROID_GLAND_EXALLC | 2859 | 378749 | 381608 |
| Cervical cancer | FinnGen | finngen_R12_CD2_INSITU_CERVIX_UTERI_EXALLC | 3701 | 219058 | 222759 |
| Bladder cancer | FinnGen | finngen_R12_C3_BLADDER_EXALLC | 4852 | 378749 | 383601 |
| non-Hodgkin lymphoma | FinnGen | finngen_R12_C3_NONHODGKIN_EXALLC | 5225 | 378749 | 383974 |

**Supplementary Table 2. 8 SNPs associated with anxiety disorders**

| **SNP** | **Effect  allele** | **Other allele** | **Effect allele  frequency** | **Beta** | **Se** | **Pval** | **Sample  size** | **F** |
| --- | --- | --- | --- | --- | --- | --- | --- | --- |
| rs1709393 | T | C | 0.5793 | -0.151 | 0.027 | 1.65E-08 | 22890 | 31.942 |
| rs2146346 | A | G | 0.5869 | 0.144 | 0.030 | 1.38E-06 | 22890 | 23.383 |
| rs2753188 | A | G | 0.7268 | 0.160 | 0.033 | 1.42E-06 | 22890 | 23.313 |
| rs112205280 | A | G | 0.5937 | 0.353 | 0.072 | 9.03E-07 | 22890 | 24.131 |
| rs58990403 | A | G | 0.792 | -0.185 | 0.039 | 2.16E-06 | 22890 | 22.411 |
| rs28373923 | A | G | 0.0675 | 0.419 | 0.092 | 4.56E-06 | 22890 | 20.999 |
| rs2740360 | T | C | 0.4743 | 0.170 | 0.033 | 3.81E-07 | 22890 | 25.785 |
| rs739315 | A | G | 0.5688 | -0.154 | 0.033 | 3.03E-06 | 22890 | 21.825 |

**Supplementary Table 3. 7 SNPs associated with lung cancer**

| **SNP** | **Effect  allele** | **Other allele** | **Effect allele  frequency** | **Beta** | **Se** | **Pval** | **Sample  size** | **F** |
| --- | --- | --- | --- | --- | --- | --- | --- | --- |
| rs37003 | C | A | 0.438937 | -0.101 | 0.015 | 3.45E-11 | 388388 | 43.901 |
| rs2526680 | T | C | 0.192059 | -0.110 | 0.019 | 1.54E-08 | 388388 | 31.996 |
| rs11571815 | A | G | 0.0091841 | 0.412 | 0.072 | 1.16E-08 | 388388 | 32.561 |
| rs12914385 | T | C | 0.35713 | 0.227 | 0.015 | 8.60E-50 | 388388 | 220.100 |
| rs8066706 | C | T | 0.0172481 | 0.331 | 0.054 | 1.00E-09 | 388388 | 37.321 |
| rs56113850 | C | T | 0.564932 | 0.092 | 0.015 | 2.35E-09 | 388388 | 35.660 |
| rs6011779 | T | C | 0.756029 | -0.116 | 0.017 | 1.31E-11 | 388388 | 45.796 |

**Supplementary Table 4. 68 SNPs associated with breast cancer**

| **SNP** | **Effect  allele** | **Other allele** | **Effect allele  frequency** | **Beta** | **Se** | **Pval** | **Sample  size** | **F** |
| --- | --- | --- | --- | --- | --- | --- | --- | --- |
| rs616488 | G | A | 0.335015 | -0.068 | 0.011 | 2.36E-10 | 246348 | 40.141 |
| rs11249433 | G | A | 0.344872 | 0.062 | 0.010 | 3.01E-09 | 246348 | 35.179 |
| rs11205277 | G | A | 0.345075 | 0.071 | 0.011 | 2.09E-11 | 246348 | 44.887 |
| rs4149909 | G | A | 0.0318199 | 0.216 | 0.028 | 5.38E-15 | 246348 | 61.118 |
| rs7594432 | C | T | 0.510035 | 0.073 | 0.010 | 3.00E-13 | 246348 | 53.205 |
| rs72827487 | T | C | 0.180116 | -0.080 | 0.013 | 1.73E-09 | 246348 | 36.253 |
| rs2016394 | A | G | 0.495826 | -0.058 | 0.010 | 7.00E-09 | 246348 | 33.535 |
| rs10197246 | C | T | 0.682909 | -0.082 | 0.011 | 2.49E-14 | 246348 | 58.102 |
| rs6721996 | A | G | 0.47357 | -0.121 | 0.010 | 1.68E-33 | 246348 | 145.493 |
| rs72953532 | T | C | 0.0276924 | -0.179 | 0.032 | 2.29E-08 | 246348 | 31.230 |
| rs6436017 | G | A | 0.485699 | 0.058 | 0.010 | 6.45E-09 | 246348 | 33.693 |
| rs41276515 | C | T | 0.272918 | 0.106 | 0.011 | 3.02E-21 | 246348 | 89.533 |
| rs28502453 | A | G | 0.129076 | -0.119 | 0.015 | 8.70E-15 | 246348 | 60.171 |
| rs17611291 | C | G | 0.601314 | -0.061 | 0.010 | 2.59E-09 | 246348 | 35.468 |
| rs10941679 | G | A | 0.265993 | 0.130 | 0.011 | 3.12E-31 | 246348 | 135.110 |
| rs12697152 | T | C | 0.768165 | -0.115 | 0.012 | 8.77E-23 | 246348 | 96.535 |
| rs112096918 | T | C | 0.199271 | -0.071 | 0.013 | 2.22E-08 | 246348 | 31.295 |
| rs1432679 | T | C | 0.595899 | -0.057 | 0.010 | 2.00E-08 | 246348 | 31.499 |
| rs76183814 | G | C | 0.0375867 | -0.165 | 0.027 | 1.95E-09 | 246348 | 36.020 |
| rs73412013 | C | A | 0.0538893 | -0.142 | 0.023 | 1.00E-09 | 246348 | 37.325 |
| rs405447 | G | A | 0.578795 | -0.067 | 0.010 | 4.55E-11 | 246348 | 43.361 |
| rs6900157 | C | T | 0.216082 | 0.098 | 0.012 | 5.02E-16 | 246348 | 65.788 |
| rs68056147 | A | G | 0.236509 | 0.075 | 0.012 | 1.62E-10 | 246348 | 40.879 |
| rs13365225 | G | A | 0.138331 | -0.083 | 0.015 | 1.91E-08 | 246348 | 31.587 |
| rs966836 | G | T | 0.795067 | 0.146 | 0.013 | 1.11E-30 | 246348 | 132.586 |
| rs55746746 | C | T | 0.556219 | 0.098 | 0.010 | 4.93E-22 | 246348 | 93.116 |
| rs7028268 | A | G | 0.391062 | -0.075 | 0.010 | 4.17E-13 | 246348 | 52.562 |
| rs10760995 | A | C | 0.384194 | 0.056 | 0.010 | 4.83E-08 | 246348 | 29.783 |
| rs4978467 | G | T | 0.156257 | 0.095 | 0.014 | 3.66E-12 | 246348 | 48.300 |
| rs10816625 | G | A | 0.0634332 | 0.231 | 0.020 | 1.45E-31 | 246348 | 136.628 |
| rs548980 | T | C | 0.662231 | 0.120 | 0.011 | 2.56E-29 | 246348 | 126.365 |
| rs2900129 | A | G | 0.144366 | -0.093 | 0.015 | 1.64E-10 | 246348 | 40.858 |
| rs10760444 | A | G | 0.530428 | -0.064 | 0.010 | 1.42E-10 | 246348 | 41.140 |
| rs12253527 | A | G | 0.316707 | 0.078 | 0.011 | 5.00E-13 | 246348 | 52.206 |
| rs10741007 | T | C | 0.962974 | 0.152 | 0.027 | 2.61E-08 | 246348 | 30.974 |
| rs10995190 | A | G | 0.201969 | -0.121 | 0.013 | 3.48E-21 | 246348 | 89.250 |
| rs72834749 | G | A | 0.121259 | -0.093 | 0.016 | 3.53E-09 | 246348 | 34.870 |
| rs1268974 | G | A | 0.663227 | -0.059 | 0.011 | 2.58E-08 | 246348 | 30.998 |
| rs11199913 | T | A | 0.392947 | -0.069 | 0.010 | 3.70E-11 | 246348 | 43.766 |
| rs11599804 | A | G | 0.392475 | 0.197 | 0.010 | 9.89E-84 | 246348 | 375.868 |
| rs688601 | A | T | 0.606559 | -0.084 | 0.010 | 3.90E-16 | 246348 | 66.286 |
| rs10896437 | C | G | 0.346283 | 0.065 | 0.011 | 7.13E-10 | 246348 | 37.985 |
| rs149796839 | G | A | 0.0163387 | 0.240 | 0.038 | 3.02E-10 | 246348 | 39.659 |
| rs78540526 | T | C | 0.0882604 | 0.306 | 0.017 | 2.79E-75 | 246348 | 337.058 |
| rs10894076 | T | C | 0.518975 | 0.062 | 0.010 | 9.41E-10 | 246348 | 37.443 |
| rs11049294 | C | G | 0.113338 | -0.141 | 0.016 | 4.46E-18 | 246348 | 75.107 |
| rs17356907 | G | A | 0.350332 | -0.097 | 0.011 | 6.29E-20 | 246348 | 83.526 |
| rs78693353 | A | C | 0.0820895 | -0.134 | 0.019 | 1.37E-12 | 246348 | 50.230 |
| rs1292010 | G | A | 0.38758 | -0.077 | 0.010 | 1.31E-13 | 246348 | 54.841 |
| rs2236007 | A | G | 0.172938 | -0.094 | 0.013 | 3.20E-12 | 246348 | 48.563 |
| rs141232262 | G | A | 0.103106 | 0.101 | 0.016 | 4.96E-10 | 246348 | 38.695 |
| rs36028293 | A | G | 0.245886 | -0.097 | 0.012 | 2.44E-16 | 246348 | 67.207 |
| rs8037137 | C | T | 0.155746 | -0.085 | 0.014 | 2.05E-09 | 246348 | 35.928 |
| rs74817977 | T | C | 0.00735869 | 0.329 | 0.055 | 2.11E-09 | 246348 | 35.865 |
| rs4784227 | T | C | 0.251759 | 0.224 | 0.011 | 6.00E-89 | 246348 | 399.829 |
| rs17817964 | T | C | 0.399943 | -0.071 | 0.010 | 4.52E-12 | 246348 | 47.884 |
| rs11650970 | T | G | 0.102599 | -0.097 | 0.017 | 1.17E-08 | 246348 | 32.537 |
| rs2787481 | C | T | 0.269774 | -0.081 | 0.011 | 9.23E-13 | 246348 | 51.001 |
| rs12964926 | A | G | 0.62927 | -0.063 | 0.010 | 1.08E-09 | 246348 | 37.174 |
| rs9952872 | C | T | 0.0685856 | -0.122 | 0.021 | 2.93E-09 | 246348 | 35.233 |
| rs2423573 | A | G | 0.332666 | -0.059 | 0.011 | 3.70E-08 | 246348 | 30.301 |
| rs2823106 | C | T | 0.27194 | -0.095 | 0.011 | 9.15E-17 | 246348 | 69.144 |
| rs12481862 | T | G | 0.0179784 | 0.204 | 0.036 | 1.78E-08 | 246348 | 31.720 |
| rs9625434 | C | T | 0.0659647 | 0.108 | 0.020 | 4.85E-08 | 246348 | 29.775 |
| rs62235753 | T | C | 0.0117967 | 0.652 | 0.040 | 7.14E-59 | 246348 | 261.749 |
| rs73156770 | A | G | 0.158933 | 0.077 | 0.014 | 2.46E-08 | 246348 | 31.092 |
| rs34623833 | A | G | 0.0993036 | 0.094 | 0.017 | 1.30E-08 | 246348 | 32.330 |
| rs12628403 | C | A | 0.10031 | 0.116 | 0.017 | 2.62E-12 | 246348 | 48.954 |

**Supplementary Table 5. 21 SNPs associated with colorectal cancer**

| **SNP** | **Effect  allele** | **Other allele** | **Effect allele  frequency** | **Beta** | **Se** | **Pval** | **Sample  size** | **F** |
| --- | --- | --- | --- | --- | --- | --- | --- | --- |
| rs28605759 | A | G | 0.537698 | -0.078 | 0.014 | 1.08E-08 | 390539 | 32.691 |
| rs12026659 | A | G | 0.250072 | 0.111 | 0.016 | 9.92E-13 | 390539 | 50.859 |
| rs7570971 | A | C | 0.408852 | 0.083 | 0.014 | 2.90E-09 | 390539 | 35.250 |
| rs73069855 | G | A | 0.107422 | 0.118 | 0.021 | 3.44E-08 | 390539 | 30.441 |
| rs4975539 | G | A | 0.0939635 | 0.173 | 0.023 | 1.75E-14 | 390539 | 58.798 |
| rs16892766 | C | A | 0.120419 | 0.155 | 0.020 | 1.00E-14 | 390539 | 59.889 |
| rs10808556 | T | C | 0.567508 | -0.132 | 0.014 | 2.91E-22 | 390539 | 94.156 |
| rs1325872 | A | G | 0.308542 | -0.094 | 0.015 | 1.91E-10 | 390539 | 40.556 |
| rs1035209 | T | C | 0.16086 | 0.133 | 0.018 | 7.51E-14 | 390539 | 55.931 |
| rs7947391 | G | A | 0.656452 | -0.088 | 0.014 | 4.28E-10 | 390539 | 38.981 |
| rs6592590 | T | C | 0.389203 | 0.081 | 0.014 | 5.05E-09 | 390539 | 34.169 |
| rs11213825 | A | C | 0.231046 | 0.120 | 0.016 | 3.47E-14 | 390539 | 57.446 |
| rs12818766 | A | G | 0.251717 | 0.102 | 0.016 | 5.50E-11 | 390539 | 42.989 |
| rs12815839 | C | T | 0.30511 | 0.082 | 0.015 | 1.60E-08 | 390539 | 31.931 |
| rs16969681 | T | C | 0.0833802 | 0.216 | 0.023 | 1.87E-20 | 390539 | 85.922 |
| rs4939567 | A | G | 0.50601 | -0.134 | 0.014 | 3.76E-23 | 390539 | 98.212 |
| rs73585909 | G | A | 0.135043 | -0.167 | 0.021 | 5.38E-16 | 390539 | 65.653 |
| rs71338563 | T | C | 0.305233 | 0.090 | 0.015 | 5.66E-10 | 390539 | 38.435 |
| rs6140071 | T | C | 0.333637 | 0.081 | 0.014 | 1.24E-08 | 390539 | 32.417 |
| rs6012932 | A | T | 0.464557 | -0.075 | 0.014 | 3.62E-08 | 390539 | 30.342 |
| rs62199217 | A | G | 0.204823 | -0.147 | 0.017 | 2.29E-17 | 390539 | 71.879 |

**Supplementary Table 6. 78SNPs associated with prostate cancer**

| **SNP** | **Effect  allele** | **Other allele** | **Effect allele  frequency** | **Beta** | **Se** | **Pval** | **Sample  size** | **F** |
| --- | --- | --- | --- | --- | --- | --- | --- | --- |
| rs35638741 | C | A | 0.663677 | -0.077 | 0.012 | 3.87E-10 | 177039 | 39.176 |
| rs111689685 | A | G | 0.265486 | 0.096 | 0.013 | 1.82E-13 | 177039 | 54.192 |
| rs141845046 | T | C | 0.0705644 | -0.151 | 0.024 | 2.63E-10 | 177039 | 39.935 |
| rs2759284 | C | T | 0.882205 | 0.105 | 0.019 | 1.79E-08 | 177039 | 31.715 |
| rs11903800 | A | G | 0.221243 | 0.084 | 0.014 | 1.20E-09 | 177039 | 36.967 |
| rs10193919 | T | C | 0.332402 | 0.071 | 0.012 | 7.78E-09 | 177039 | 33.329 |
| rs62137468 | C | G | 0.343402 | 0.072 | 0.012 | 4.53E-09 | 177039 | 34.381 |
| rs1038822 | C | T | 0.655595 | -0.105 | 0.012 | 6.68E-18 | 177039 | 74.309 |
| rs2539981 | C | T | 0.488958 | 0.118 | 0.012 | 7.28E-24 | 177039 | 101.463 |
| rs146441981 | C | A | 0.0471375 | 0.167 | 0.027 | 8.37E-10 | 177039 | 37.671 |
| rs116532339 | G | T | 0.0439799 | -0.249 | 0.030 | 8.48E-17 | 177039 | 69.295 |
| rs77559646 | A | G | 0.028573 | 0.251 | 0.033 | 3.84E-14 | 177039 | 57.248 |
| rs77482050 | A | G | 0.054929 | -0.329 | 0.028 | 7.18E-33 | 177039 | 142.602 |
| rs6788616 | A | G | 0.526842 | -0.098 | 0.012 | 5.66E-17 | 177039 | 70.092 |
| rs1283106 | A | C | 0.391663 | 0.072 | 0.012 | 1.34E-09 | 177039 | 36.747 |
| rs1567780 | C | T | 0.486899 | -0.069 | 0.012 | 2.61E-09 | 177039 | 35.458 |
| rs7641133 | C | T | 0.706038 | -0.112 | 0.013 | 9.24E-19 | 177039 | 78.215 |
| rs78416326 | C | G | 0.21225 | -0.166 | 0.015 | 4.14E-30 | 177039 | 129.978 |
| rs344140 | C | G | 0.699196 | -0.075 | 0.013 | 4.11E-09 | 177039 | 34.571 |
| rs2452578 | G | A | 0.276076 | -0.094 | 0.013 | 8.71E-13 | 177039 | 51.115 |
| rs10007915 | G | C | 0.488915 | -0.111 | 0.012 | 2.66E-21 | 177039 | 89.781 |
| rs2242652 | A | G | 0.228998 | -0.141 | 0.014 | 2.13E-23 | 177039 | 99.333 |
| rs75630086 | C | G | 0.0741649 | 0.138 | 0.022 | 2.39E-10 | 177039 | 40.123 |
| rs530543670 | A | G | 0.417917 | 0.140 | 0.012 | 4.03E-32 | 177039 | 139.176 |
| rs138485750 | C | T | 0.0355848 | -0.230 | 0.033 | 6.01E-12 | 177039 | 47.327 |
| rs145315307 | G | T | 0.0379326 | -0.280 | 0.033 | 1.26E-17 | 177039 | 73.055 |
| rs6935446 | G | A | 0.350804 | 0.105 | 0.012 | 5.08E-18 | 177039 | 74.848 |
| rs2782299 | A | C | 0.647472 | 0.089 | 0.012 | 5.21E-13 | 177039 | 52.123 |
| rs9371670 | G | C | 0.388985 | -0.067 | 0.012 | 3.34E-08 | 177039 | 30.499 |
| rs2774218 | A | G | 0.813753 | -0.102 | 0.015 | 4.30E-12 | 177039 | 47.982 |
| rs12666766 | A | C | 0.666502 | -0.072 | 0.012 | 5.87E-09 | 177039 | 33.879 |
| rs10486567 | A | G | 0.261063 | -0.145 | 0.014 | 1.04E-26 | 177039 | 114.450 |
| rs11977526 | A | G | 0.324747 | 0.078 | 0.012 | 3.16E-10 | 177039 | 39.570 |
| rs11764933 | A | G | 0.475381 | -0.084 | 0.012 | 4.95E-13 | 177039 | 52.225 |
| rs13272392 | A | T | 0.508075 | -0.151 | 0.012 | 7.56E-39 | 177039 | 169.956 |
| rs182955053 | C | A | 0.0714766 | 0.171 | 0.022 | 7.02E-15 | 177039 | 60.593 |
| rs4870990 | G | A | 0.686497 | 0.140 | 0.013 | 1.14E-28 | 177039 | 123.404 |
| rs73351629 | G | C | 0.289108 | -0.196 | 0.013 | 5.47E-52 | 177039 | 230.174 |
| rs62529864 | T | C | 0.00904368 | -0.460 | 0.069 | 2.02E-11 | 177039 | 44.950 |
| rs77541621 | A | G | 0.0256266 | 0.595 | 0.033 | 4.36E-71 | 177039 | 317.803 |
| rs6983267 | T | G | 0.474052 | -0.202 | 0.012 | 1.61E-67 | 177039 | 301.434 |
| rs7812894 | T | A | 0.828748 | -0.399 | 0.015 | 1.55E-162 | 177039 | 738.113 |
| rs10993994 | G | A | 0.650553 | -0.164 | 0.012 | 5.52E-41 | 177039 | 179.738 |
| rs12262998 | T | C | 0.356025 | -0.083 | 0.012 | 9.58E-12 | 177039 | 46.413 |
| rs7907606 | G | T | 0.136789 | 0.094 | 0.017 | 2.11E-08 | 177039 | 31.391 |
| rs11199869 | G | A | 0.274023 | -0.092 | 0.013 | 3.61E-12 | 177039 | 48.328 |
| rs6579002 | G | A | 0.759558 | -0.148 | 0.013 | 4.46E-28 | 177039 | 120.692 |
| rs139082459 | A | C | 0.0323093 | 0.181 | 0.032 | 1.30E-08 | 177039 | 32.335 |
| rs12278923 | C | A | 0.478482 | 0.174 | 0.012 | 2.43E-50 | 177039 | 222.617 |
| rs12285347 | C | T | 0.409606 | -0.071 | 0.012 | 2.23E-09 | 177039 | 35.757 |
| rs3217810 | T | C | 0.15317 | 0.090 | 0.016 | 2.27E-08 | 177039 | 31.251 |
| rs644524 | A | G | 0.427007 | -0.070 | 0.012 | 6.53E-09 | 177039 | 33.672 |
| rs73106451 | A | C | 0.157264 | 0.167 | 0.016 | 1.32E-26 | 177039 | 113.976 |
| rs1405778 | G | A | 0.620667 | 0.069 | 0.012 | 1.04E-08 | 177039 | 32.757 |
| rs2555009 | G | A | 0.565325 | -0.096 | 0.012 | 2.51E-16 | 177039 | 67.152 |
| rs7295014 | A | G | 0.581227 | -0.072 | 0.012 | 1.00E-09 | 177039 | 37.323 |
| rs7336001 | C | G | 0.0818262 | -0.170 | 0.022 | 1.45E-14 | 177039 | 59.168 |
| rs1955750 | T | C | 0.584752 | 0.072 | 0.012 | 1.45E-09 | 177039 | 36.598 |
| rs145988452 | C | T | 0.0424935 | -0.239 | 0.031 | 7.92E-15 | 177039 | 60.354 |
| rs62003539 | C | T | 0.138451 | -0.126 | 0.017 | 2.43E-13 | 177039 | 53.622 |
| rs12952830 | A | G | 0.0628121 | 0.145 | 0.023 | 4.43E-10 | 177039 | 38.915 |
| rs62075729 | T | C | 0.0294672 | -0.209 | 0.037 | 1.00E-08 | 177039 | 32.839 |
| rs3110641 | A | G | 0.215054 | 0.094 | 0.014 | 2.72E-11 | 177039 | 44.368 |
| rs11651755 | C | T | 0.383078 | -0.199 | 0.012 | 5.46E-61 | 177039 | 271.460 |
| rs145138440 | T | C | 0.00806002 | 0.611 | 0.059 | 4.58E-25 | 177039 | 106.945 |
| rs2303488 | T | C | 0.195155 | 0.100 | 0.015 | 6.00E-12 | 177039 | 47.331 |
| rs145922598 | T | C | 0.013426 | 1.039 | 0.042 | 1.60E-133 | 177039 | 604.692 |
| rs6504581 | A | G | 0.136685 | 0.163 | 0.017 | 4.86E-23 | 177039 | 97.702 |
| rs78059576 | A | C | 0.00603265 | 1.085 | 0.062 | 1.31E-69 | 177039 | 311.027 |
| rs113269929 | A | G | 0.00927783 | 0.354 | 0.056 | 1.79E-10 | 177039 | 40.679 |
| rs8072254 | G | A | 0.49902 | -0.102 | 0.012 | 2.11E-18 | 177039 | 76.587 |
| rs12610267 | G | A | 0.59494 | -0.131 | 0.012 | 1.34E-28 | 177039 | 123.079 |
| rs8112363 | C | T | 0.279945 | -0.098 | 0.013 | 1.30E-13 | 177039 | 54.848 |
| rs17632542 | C | T | 0.0677218 | -0.270 | 0.024 | 2.00E-29 | 177039 | 126.851 |
| rs2777939 | T | C | 0.753203 | 0.122 | 0.014 | 6.76E-19 | 177039 | 78.834 |
| rs114703330 | C | T | 0.0707802 | -0.153 | 0.024 | 7.38E-11 | 177039 | 42.416 |
| rs62237573 | T | C | 0.0166147 | 0.317 | 0.044 | 9.44E-13 | 177039 | 50.956 |
| rs73179053 | C | T | 0.0641618 | -0.163 | 0.025 | 2.87E-11 | 177039 | 44.263 |

**Supplementary Table 7. 16SNPs associated with thyroid cancer**

| **SNP** | **Effect  allele** | **Other allele** | **Effect allele  frequency** | **Beta** | **Se** | **Pval** | **Sample  size** | **F** |
| --- | --- | --- | --- | --- | --- | --- | --- | --- |
| rs675763 | G | A | 0.598628 | 0.174 | 0.027 | 1.89E-10 | 381608 | 40.581 |
| rs12061451 | A | C | 0.294377 | 0.161 | 0.028 | 1.53E-08 | 381608 | 32.021 |
| rs11693806 | G | C | 0.694432 | -0.271 | 0.028 | 1.53E-22 | 381608 | 95.431 |
| rs4975538 | C | G | 0.408057 | 0.165 | 0.027 | 7.96E-10 | 381608 | 37.770 |
| rs17134152 | T | G | 0.139855 | -0.234 | 0.041 | 8.00E-09 | 381608 | 33.274 |
| rs62401231 | G | A | 0.312225 | 0.161 | 0.028 | 1.10E-08 | 381608 | 32.663 |
| rs2572440 | T | G | 0.603338 | 0.160 | 0.028 | 7.24E-09 | 381608 | 33.468 |
| rs112852637 | C | T | 0.566499 | 0.247 | 0.027 | 6.68E-20 | 381608 | 83.405 |
| rs13287517 | C | G | 0.341083 | 0.175 | 0.027 | 1.86E-10 | 381608 | 40.607 |
| rs10759944 | G | A | 0.652068 | -0.429 | 0.026 | 4.02E-60 | 381608 | 267.480 |
| rs12237985 | A | T | 0.169463 | 0.237 | 0.034 | 2.63E-12 | 381608 | 48.944 |
| rs67549948 | G | A | 0.952253 | -0.377 | 0.056 | 2.49E-11 | 381608 | 44.544 |
| rs2780310 | G | A | 0.425482 | -0.193 | 0.027 | 1.02E-12 | 381608 | 50.809 |
| rs72743467 | C | A | 0.22259 | 0.209 | 0.031 | 1.15E-11 | 381608 | 46.048 |
| rs2013832 | A | G | 0.0649318 | 0.325 | 0.049 | 3.80E-11 | 381608 | 43.713 |
| rs62235753 | T | C | 0.0110359 | 0.943 | 0.095 | 2.06E-23 | 381608 | 99.399 |

**Supplementary Table 8. 5SNPs associated with cervical cancer**

| **SNP** | **Effect  allele** | **Other allele** | **Effect allele  frequency** | **Beta** | **Se** | **Pval** | **Sample  size** | **F** |
| --- | --- | --- | --- | --- | --- | --- | --- | --- |
| rs27069 | T | C | 0.417358 | -0.153 | 0.024 | 1.76E-10 | 222759 | 40.712 |
| rs2266161 | A | G | 0.120063 | -0.231 | 0.038 | 1.32E-09 | 222759 | 36.783 |
| rs77114005 | C | T | 0.258714 | -0.195 | 0.028 | 1.48E-12 | 222759 | 50.079 |
| rs28479464 | C | T | 0.293702 | -0.275 | 0.027 | 5.94E-25 | 222759 | 106.427 |
| rs3129197 | A | G | 0.161942 | 0.185 | 0.031 | 1.31E-09 | 222759 | 36.805 |

**Supplementary Table 9. 9SNPs associated with bladder cancer**

| **SNP** | **Effect  allele** | **Other allele** | **Effect allele  frequency** | **Beta** | **Se** | **Pval** | **Sample  size** | **F** |
| --- | --- | --- | --- | --- | --- | --- | --- | --- |
| rs17863783 | T | G | 0.0374096 | -0.476 | 0.063 | 5.63E-14 | 383601 | 56.495 |
| rs10069690 | T | C | 0.294995 | -0.185 | 0.024 | 3.31E-15 | 383601 | 62.073 |
| rs72826303 | A | G | 0.371243 | 0.128 | 0.022 | 3.11E-09 | 383601 | 35.113 |
| rs10094872 | T | A | 0.448471 | 0.245 | 0.021 | 2.68E-31 | 383601 | 135.417 |
| rs2976398 | C | G | 0.514498 | 0.183 | 0.021 | 4.65E-18 | 383601 | 75.024 |
| rs372042 | C | G | 0.208844 | 0.151 | 0.026 | 1.08E-08 | 383601 | 32.689 |
| rs28397975 | A | T | 0.737028 | -0.131 | 0.024 | 2.91E-08 | 383601 | 30.765 |
| rs2161516 | T | C | 0.35997 | 0.125 | 0.022 | 1.10E-08 | 383601 | 32.649 |
| rs5750711 | C | T | 0.439621 | -0.120 | 0.021 | 1.79E-08 | 383601 | 31.707 |

**Supplementary Table 10. 10SNPs associated with non-Hodgkin lymphoma**

| **SNP** | **Effect  allele** | **Other allele** | **Effect allele  frequency** | **Beta** | **Se** | **Pval** | **Sample  size** | **F** |
| --- | --- | --- | --- | --- | --- | --- | --- | --- |
| rs3806624 | G | A | 0.478053 | 0.128 | 0.020 | 1.40E-10 | 383974 | 41.168 |
| rs669607 | C | A | 0.425839 | 0.110 | 0.020 | 3.27E-08 | 383974 | 30.538 |
| rs542887 | T | C | 0.388429 | 0.114 | 0.020 | 1.71E-08 | 383974 | 31.794 |
| rs76106586 | G | A | 0.0141289 | 0.462 | 0.075 | 5.99E-10 | 383974 | 38.326 |
| rs4348357 | G | T | 0.624185 | -0.127 | 0.020 | 3.63E-10 | 383974 | 39.300 |
| rs4713570 | T | C | 0.293355 | 0.249 | 0.021 | 3.14E-32 | 383974 | 139.670 |
| rs13255292 | T | C | 0.343843 | 0.151 | 0.021 | 2.07E-13 | 383974 | 53.940 |
| rs40836 | G | A | 0.527445 | 0.132 | 0.020 | 3.52E-11 | 383974 | 43.865 |
| rs2029745 | C | T | 0.425449 | 0.134 | 0.020 | 2.65E-11 | 383974 | 44.422 |
| rs6032662 | T | C | 0.727079 | -0.123 | 0.022 | 1.88E-08 | 383974 | 31.612 |
